# Predictive, Preventive and Personalised Medicine as the hardcore of ‘Horizon 2020’: EPMA position paper

**DOI:** 10.1186/1878-5085-5-6

**Published:** 2014-04-07

**Authors:** Olga Golubnitschaja, Judita Kinkorova, Vincenzo Costigliola

**Affiliations:** 1 Department of Radiology, Rheinische Friedrich-Wilhelms-University of Bonn, 53105 Bonn, Germany; 2 The European Association for Predictive, Preventive and Personalised Medicine, 1150 Brussels, Belgium; 3 Technology Centre Academy of Sciences, Prague 165 02, Czech Republic; 4 The European Medical Association, 1160 Brussels, Belgium

**Keywords:** Predictive, Preventive and Personalised Medicine, Horizon 2020, Excellent science and technological innovation, Societal challenges, Industrial leadership, Health care, Integrative bioinformatics, Economy, Ethics, Education

## Abstract

The European Association for Predictive, Preventive and Personalised Medicine (EPMA) considers acute problems in medical sciences as well as the quality and management of medical services challenging health care systems in Europe and worldwide. This actuality has motivated the representatives of EPMA to comment on the efforts in promoting an integrative approach based on multidisciplinary expertise to advance health care-related research and management. The current paper provides a global overview of the problems related to medical services: pandemic scenario in the progression of common non-communicable diseases, delayed interventional approaches of reactive medicine, poor economy of health care systems, lack of specialised educational programmes, problematic ethical aspects of several treatments as well as inadequate communication among professional groups and policymakers. In the form of individual paragraphs, the article presents a consolidated position of PPPM professionals towards the new European programme ‘Horizon 2020’ providing the long-lasting instruments for scientific and technological progress in medical services and health care-related programmes. In the author's opinion, Horizon 2020 provides unlimited room for research and implementation in Predictive, Preventive and Personalised Medicine. However, the overall success of the programme strongly depends on the effective communication and consolidation of professionals relevant for PPPM as well as the communication quality with policymakers. Smart political decision is the prerequisite of the effective PPPM implementation in the health care sector. This position is focused on the patients' needs, innovative medical sciences, optimal health and disease management, expert recommendations for the relevant medical fields and optimal solutions which have a potential to advance health care services if the long-term strategies were to be effectively implemented as proposed here.

## Predictive, Preventive and Personalised Medicine as the medicine of the future: EPMA vision

### Current health care: What is behind the issue?

For many acute and chronic disorders, the current health care outcomes are considered as being inadequate: global figures cry for preventive measures and personalised treatments. In fact, severe chronic pathologies such as cardiovascular disorders, diabetes and cancer are treated after onset of the disease, frequently at near-end stages. Pessimistic prognosis considers pandemic scenario for type 2 diabetes mellitus, neurodegenerative disorders and some types of cancer over the next 10–20 years followed by the economic disaster of health care systems in a global scale.

### Advanced health care tailored to the person: What is beyond the issue?

Advanced health care promotes the paradigm change from delayed interventional to predictive medicine tailored to the person, from reactive to preventive medicine and from disease to wellness. The innovative Predictive, Preventive and Personalised Medicine (PPPM) is emerging as the focal point of efforts in health care aimed at curbing the prevalence of both communicable and non-communicable diseases such as diabetes, cardiovascular diseases, chronic respiratory diseases, cancer and dental pathologies. The cost-effective management of diseases and the critical role of PPPM in modernisation of health care have been acknowledged as priorities by global and regional organisations and health-related institutions such as the Organisation of United Nations, the European Union and the National Institutes of Health.

### Why integrative medical approach by PPPM as the medicine of the future?

PPPM is the new integrative concept in health care sector that enables to predict individual predisposition before onset of the disease, to provide targeted preventive measures and create personalised treatment algorithms tailored to the person. The expected outcomes are conducive to more effective population screening, prevention early in childhood, identification of persons at risk, stratification of patients for the optimal therapy planning, prediction and reduction of adverse drug-drug or drug-disease interactions relying on emerging technologies, such as pharmacogenetics, pathology-specific molecular patters, subcellular imaging, disease modelling and individual patient profiles. Integrative approach by PPPM is considered as the medicine of the future. Being at the forefront of the global efforts, the European Association for Predictive, Preventive and Personalised Medicine (EPMA, http://www.epmanet.eu/) promotes the integrative concept of PPPM among health care stakeholders, governmental institutions, educators, funding bodies, patient organisations and in the public domain.

### Multidisciplinary aspects of advanced bio/medical approaches and innovative technologies overviewed by EPMA/Springer book series

**Figure 1:**
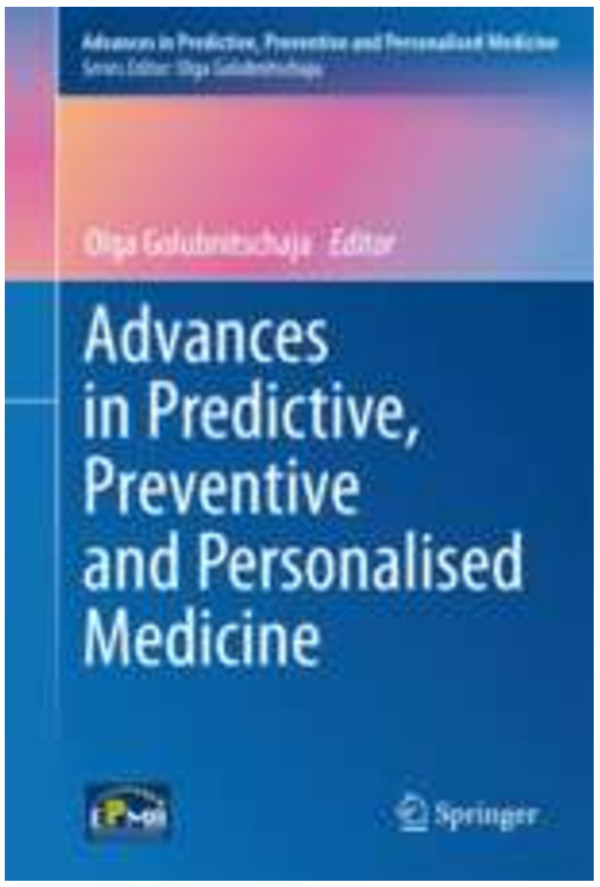
**Book series *Advances in Predictive, Preventive and Personalised Medicine*.** This is the specialised didactic material for Universities, medical centres and specialised international programmes [1].

For the paradigm shift from delayed reactive to Predictive, Preventive and Personalised Medicine, a new culture should be created in communication between individual professional domains, between doctor and patient, as well as in communication with individual social (sub)groups and patient cohorts. Considering this long-term mission, a particular role is dedicated to the multidisciplinary and trans-domain education in PPPM requiring innovative design of specialised didactic materials. In this context, an integration of a spectrum of professional groups into the overall concept of PPPM is a particular advantage of the EPMA/Springer book series *Advances in Predictive, Preventive and Personalised Medicine—*the didactic material specialised in PPPM (see Figure 1). Expert recommendations focus on the cost-effective management tailored to the person in health and disease. Innovative strategies are considered for standardisation of health care services. New guidelines are proposed for medical ethics, treatment of rare diseases, innovative approaches to early and predictive diagnostics, patient stratification and targeted prevention in healthy individuals, persons at risk, individual patient groups, subpopulations, institutions, health care economy and marketing.

The additional bibliographic details of the series are the following:

Series Editor: Olga Golubnitschaja

Released volumes:

–Healthcare Overview: New Perspectives (Ed: V. Costigliola) ISBN 978-94-007-4602-2

–New Strategies to Advance Pre/Diabetes Care: Integrative Approach by PPPM (Ed: M. Mozaffari) ISBN 978-94-007-5970-1

–Neurodegenerative Diseases: Integrative PPPM Approach as the Medicine of the Future (Ed: S. Mandel) ISBN 978-94-007-5865-0

–Drug Delivery Systems: Advanced Technologies Potentially Applicable in Personalised Treatments (Ed: J. Coelho) ISBN 978-94-007-6009-7

Volumes releasing in 2014–2015:

–Rare Diseases: Integrative PPPM Approach as the Medicine of the Future (Ed: M. Özgüç)

–Individualised Medicine: Ethical, economical and historical perspectives (Ed: T. Fischer, M. Langanke, P. Marschall);

–Preventive and Predictive Genetics: Towards Personalised Medicine (Ed: G. Grech, I. Grossman)

–Circulating nucleic acids in early diagnosis, prognosis and treatment monitoring: an introduction (Ed: P. Gahan).

## PPPM in Horizon 2020—short overview of the history and preparatory steps: the facts speak for themselves

### Basic EPMA event, November 13, 2008

The juristic protocol has been signed—the Association is born now. Its objectives are to create and promote the concepts and practical realisation of Predictive, Preventive and Personalised Medicine.

**Figure 2:**
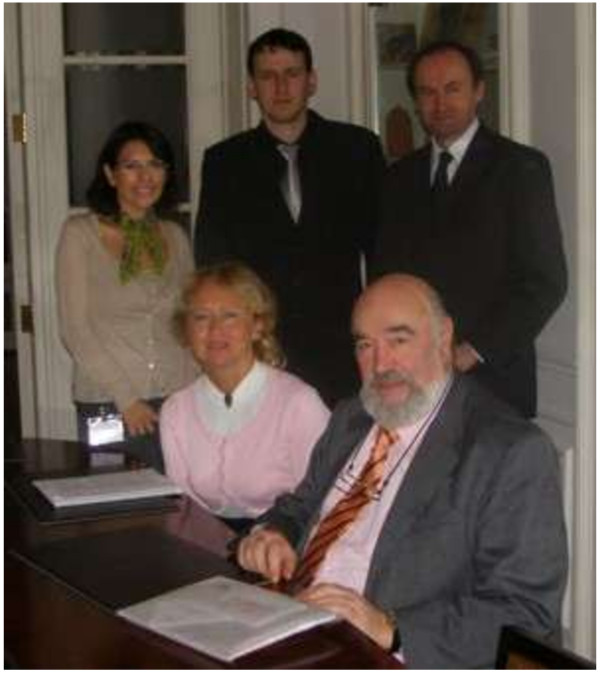
**Founder members of the Directorate.** (*from right to left*) *First line*: V. Costigliola (President), O. Golubnitschaja (Secretary General). *Second line*: K. Krapfenbauer (Vice President responsible for European affairs), M. Kapalla (Director responsible for contacts with industry), S. Mandel (Vice President responsible for associated countries and contacts apart from Europe).

The founder members of the Directorate are shown in Figure 2.

### The global concepts of Predictive, Preventive and Personalised Medicine—the medicine of the future

**Figure 3:**
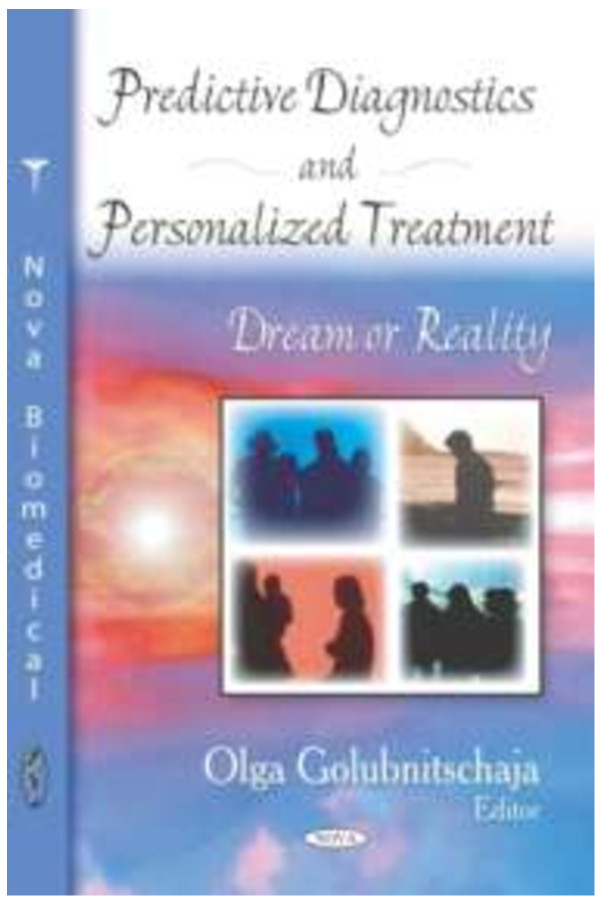
***Predictive Diagnostics and Personalized Treatment: Dream or Reality*.** Editor: O. Golubnitschaja, ‘Nova Science Publishers’, New York, June 2009.

The following important aspects are covered in the book *Predictive Diagnostics and Personalized Treatment: Dream or Reality*, edited by O. Golubnitschaja published in 2009 (see Figure 3):

•Predictive Medicine as the new philosophy in health care.

•Advanced technologies in Predictive and Personalised Medicine.

•Prediction in non-diseased population.

•Targeted preventive measures.

•Prediction of secondary complications.

•Prediction of drug sensitivity and drug response.

•Personalised patient treatment.

•Optimised therapy planning.

•Role of information systems.

•Economic aspects.

•Role of insurance.

•Ethics.

### First meeting of EPMA representatives with the Vice Secretary of UNO

**Figure 4:**
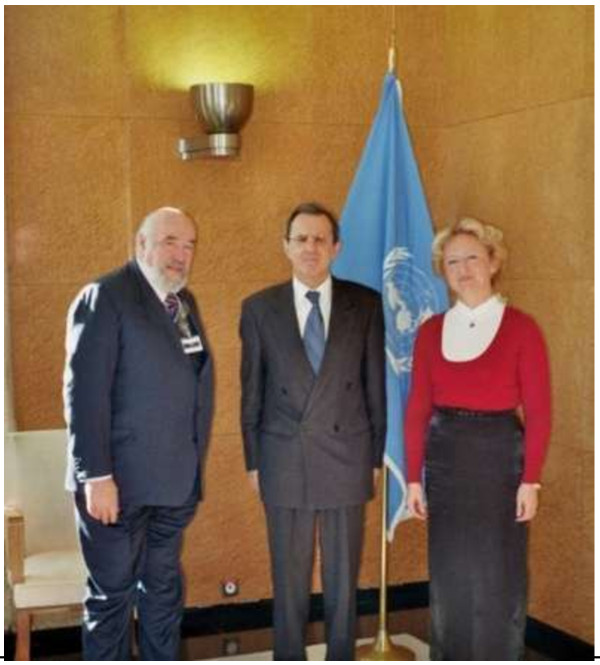
**EPMA goes global in consensus with United Nations.***Left to right:* EPMA President, V. Costigliola, UNO Vice Secretary General S. Ordzhonikidze, EPMA Secretary General O. Golubnitschaja.

The first meeting of EPMA representatives with the Vice Secretary of UNO took place in Geneva on December 8, 2009 (see Figure 4).

### Publication of specialised issue of *The EPMA Journal* on global PPPM concepts for stakeholders

**Figure 5:**
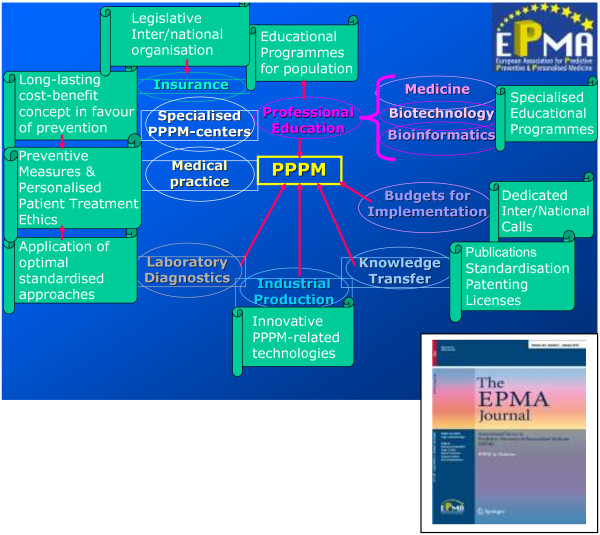
**Global PPPM concepts for stakeholders, *The EPMA Journal 2010*, Springer.**

Global PPPM concepts for stakeholders (researchers, health care providers, patient organisations, policymakers) have been published by the specialised issue of *The EPMA Journal* in March 2010 [2] (Figure 5).

### Organisation of a series of preparatory workshops to create Horizon 2020 by EU Commission

**Figure 6:**
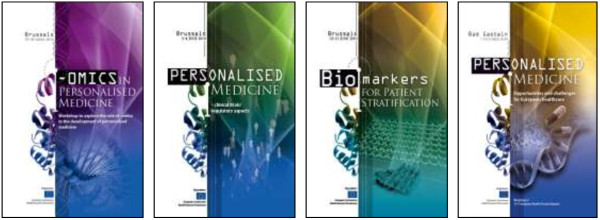
**A series of the preparatory workshops by the EU-Commission in Brussels in order to create Horizon 2020.** (*from left to right*) *OMICS in Personalised Medicine (workshop to explore the role of *omics in the development of personalised medicine); Clinical trials/regulatory aspects; Biomarkers for patient stratification; Opportunities and challenges for European health care.

In the year 2010, the European Commission has organised a series of preparatory workshops in order to create Horizon 2020 (Figure 6). Several EPMA experts have been invited to chair, talk and create protocols at the sessions [3].

### Final conference in preparation of Horizon 2020 by the EU Commission

**Figure 7:**
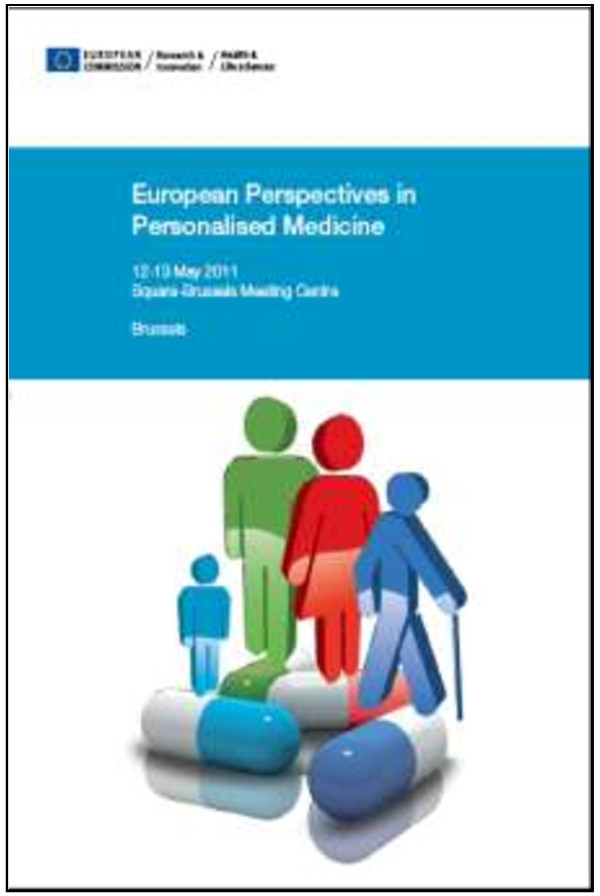
European event of high importance, May 2011.

The finalising conference in the preparation of Horizon 2020 by the EU Commission took place in Brussels on May 12–13, 2011 (see Figure 7) [4]. Among the invited speakers were

–Prof. Dr. R. Danesi, The National EPMA Representative in Italy.

–Prof. Dr. C. Swanton, The member of the National EPMA BOARD in UK.

–Prof. Dr. O. Golubnitschaja, Editor-in-Chief and Secretary General of EPMA.

### EPMA World Congress

**Figure 8:**
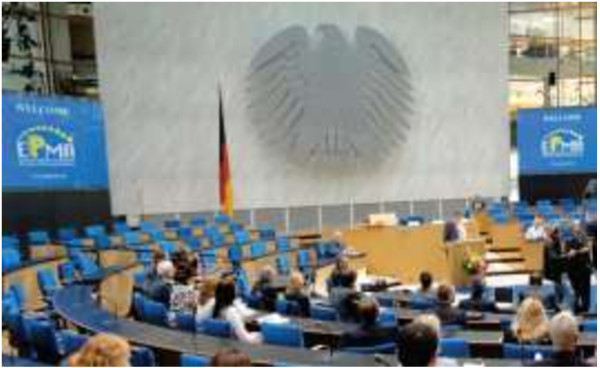
**Bonn, Germany, September 2011.** Over 40 countries have been represented at the EPMA World Congress in PPPM.

In September 2011, EPMA World Congress took place in Bonn, Germany to discuss and create long-term strategies in PPPM (see Figure 8).

### Release of the White Paper 2012, *The EPMA Journal*

Consequently, EPMA has released its fundamental document**—**White Paper 2012 of the European Association for Predictive, Preventive and Personalised Medicine, *The EPMA Journal* in 2012, *Open Access*[5].

### EPMA Summit

**Figure 9:**
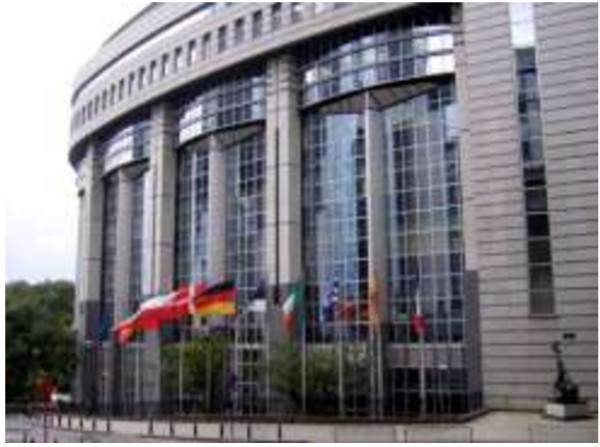
Brussels, September 2013: EPMA Summit in the EU Parliament.

Finally, the EPMA Summit took place in the EU Parliament to discuss PPPM-relevant programmes in Horizon 2020, professional consolidation and communication with policymakers [6] (see Figure 9).

### Release of *Horizon 2020—Work Programme 2014–2015*

**Figure 10:**
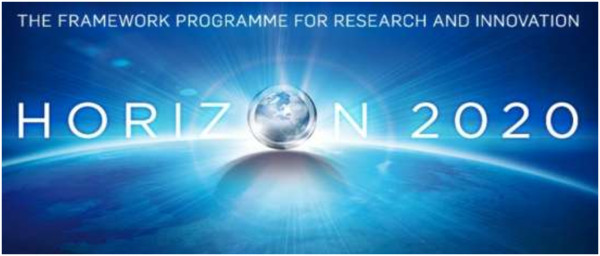
Horizon 2020 as the long-term European programme to support research and the innovation.

On December 11, 2013 the EU Commission has released *Horizon 2020—Work Programme 2014–2015* ‘Health, demographic change and well-being’ [7] (Figure 10).

## Horizon 2020 as the European long-term strategy in medicine: targets, domains, structure and overall meaning of the programme

**Figure 11:**
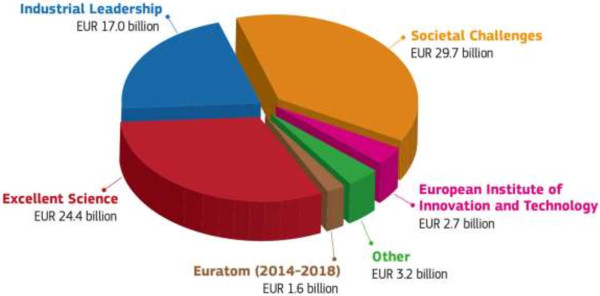
Diagram illustrates the budget distribution in Horizon 2020.

Horizon 2020 is the biggest EU Research and Innovation programme ever with the budget almost EUR 80 billion of funding for the next 7 years (2014–2020)—see Figure 11[8].

Horizon 2020 is the financial instrument implementing the Innovation Union Europe 2020 [9], a flagship initiative aimed at securing Europe's global competitiveness.

**Figure 12:**
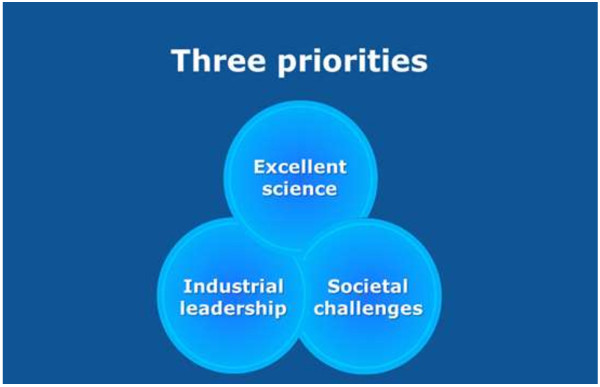
Horizon 2020 is focused on excellence in science, industrial leadership and tackling societal challenges.

Horizon 2020 follows achievements in the previous programme, 7th Framework Programme for Research and Technological Development (2007 to 2013). Horizon 2020 differs in the structure from FP7, by coupling research and innovation, aiming to achieve this with its emphasis on excellent science, industrial leadership and tackling societal challenges (see Figure 12). The goal is to ensure that Europe produces world-class science, removes barriers to innovation and makes it easier for the public and private sectors to work together in delivering innovation.

### What is new?

Horizon combines three previously separate programmes: Framework programme (FP), Competitiveness and Innovation Framework Programme (CIP), and the European Institute of Innovation and Technology (EIT).

Horizon 2020 brings several novelties. Amongst them are the following:

•Simplification through a simpler programme architecture (structure)

•Simplified access for all companies, universities, institutes in all EU countries and beyond

•Single set of rules with a simplified reimbursement procedure.

•Single point of access for participants.

Horizon 2020 is open to everyone, with a simple structure that reduces red tape and time so participants can focus on what is really important. This approach makes sure new projects get off the ground quickly and achieve results faster.

The EU Framework Programme for Research and Innovation will be complemented by further measures to complete and further develop the European Research Area. These measures will aim at breaking down barriers to create a genuine single market for knowledge, research and innovation [8].

### Structure of Horizon 2020

**Figure 13:**
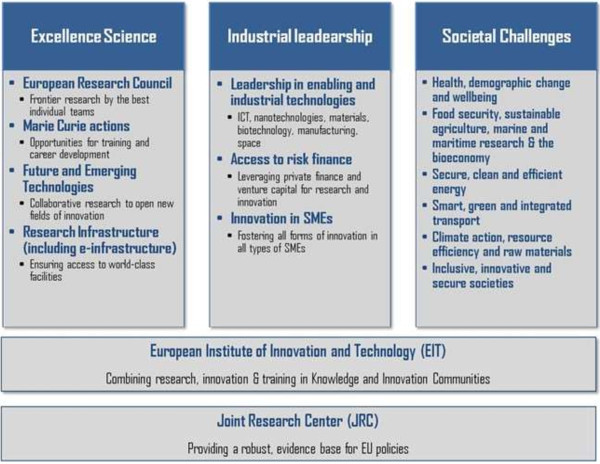
Priorities of Horizon 2020 that correspond to those of Europe 2020 and Innovation Union.

Horizon 2020 will focus resources on three distinct, yet mutually reinforcing, priorities, where there is clear Union added value. These priorities correspond to those of Europe 2020 and the Innovation Union (see Figure 13).

(1)  *Excellent Science* – This will raise the level of excellence in Europe's science base and ensure a steady stream of world-class research to secure Europe's long-term competitiveness. It will support the best ideas, develop talent within Europe, provide researchers with access to priority research infrastructure, and make Europe an attractive location for the world's best researchers. This will

•Support the most talented and creative individuals and their teams to carry out frontier research of the highest quality by building on the success of the *European Research Council.*

•Fund collaborative research to open up new and promising fields of research and innovation through support for *Future and Emerging Technologies* (FET).

•Provide researchers with excellent training and career development opportunities through the *Marie Skłodowska-Curie actions ('Marie Curie actions').*

•Ensure that Europe has world-class *research infrastructures* (including eInfrastructures) accessible to all researchers in Europe and beyond.

(2)  *Industrial Leadership* – This will aim at making Europe a more attractive location to invest in research and innovation (including eco-innovation), by promoting activities where businesses set the agenda. It will provide major investment in key industrial technologies, maximise the growth potential of European companies by providing them with adequate levels of finance and help innovative small and medium enterprises (SMEs) to grow into world-leading companies. This will

•Build *leadership in enabling and industrial technologies*, with dedicated support for informatics and communicating technologies (ICT), na2chnologies, advanced materials, biotechnology, advanced manufacturing and processing, and space, while also providing support for cross-cutting actions to capture the accumulated benefits from combining several Key Enabling Technologies.

•Facilitate *access to risk finance.*

•Provide Union wide support for *innovation in SMEs*.

(3) *Societal Challenges* – This reflects the policy priorities of the Europe 2020 strategy and addresses major concerns shared by citizens in Europe and elsewhere. A challenge-based approach will bring together resources and knowledge across different fields, technologies and disciplines, including social sciences and the humanities. This will cover activities from research to market with a new focus on innovation-related activities, such as piloting, demonstration, test beds, and support for public procurement and market uptake. It will include establishing links with the activities of the European Innovation Partnerships. Funding will be focussed on the following challenges:

•Health, demographic change and well-being.

•Food security, sustainable agriculture, marine and maritime research and the bioeconomy.

•Secure, clean and efficient energy.

•Smart, green and integrated transport.

•Climate action, resource efficiency and raw materials.

•Inclusive, innovative and secure societies.

The EIT will play an important role by combining excellent research, education and innovation, thus integrating the knowledge triangle. The EIT will do so primarily through the Knowledge and Innovation Communities (KICs). In addition, it will ensure that experiences are shared beyond the KICs through targeted dissemination and knowledge-sharing measures.

The Joint Research Centre's activities will be an integral part of Horizon 2020, providing robust, evidence-based support to Union policies. This will be driven by customer needs complemented by forward-looking activities.

Horizon 2020 will be a 7-year programme and there may be significant shifts in the broader economic and policy context as the programme progresses. Ensuring Horizon 2020's continued relevance will therefore also require to adjust priorities and resources, as and when necessary. As such, flexibility clauses have been included in the proposal in this respect.

The implementation of Horizon 2020 will also take a strategic approach to programming of research and innovation, using joint actions and modes of governance aligning closely with policy development yet cutting across the boundaries of traditional sectoral policies. This will be based on sound evidence, analysis and foresight, with progress measured against a robust set of indicators.

As regards the funding of research activities involving human embryonic stem cells, the Horizon 2020 legislative package is fully in line with the approach supported by the European Parliament and the Council upon their adoption of the FP7 legislation, as set out in the Commission's statement of 2006 (OJ L412 of 30 December 2006).

### International cooperation

International cooperation with the third countries is necessary to address effectively many specific objectives defined in Horizon 2020. This is the case in particular for all the societal challenges addressed by Horizon 2020, which need to be tackled at the global level. International cooperation is also essential for frontier and basic research in order to capture the benefits from emerging science and technology opportunities. Promoting the international mobility of researchers and innovation staff is crucial for enhancing this global cooperation. Activities at the international level are equally important to enhance the competitiveness of European industry by promoting the take-up and trade of novel technologies, for instance through the development of worldwide standards and guidelines and by promoting the acceptance and deployment of European solutions outside Europe.

The aim of international cooperation in Horizon 2020 will be to strengthen the Union's excellence and attractiveness in research, to tackle global challenges jointly and to support the Union's external policies. The focus of international cooperation in Horizon 2020 will be on cooperation with three major country groupings:

(1)  Industrialised and emerging economies.

(2)  Enlargement and neighbourhood countries.

(3)  Enlargement of developing countries.

Where appropriate, Horizon 2020 will promote cooperation at the regional or multilateral level. International cooperation in research and innovation is a key aspect of the Union's global commitments and has an important role to play in the Union's partnership with developing countries, which are often disproportionately affected by global challenges. This cooperation will promote inclusive growth and progressing towards the achievement of the Millennium Development Goals and other goals agreed in the framework of international sustainable development.

Horizon 2020 will continue with the principle of general openness, while encouraging reciprocal access to third country programmes. In addition, a range of targeted actions will be implemented taking a strategic approach to international cooperation on the basis of common interest and mutual benefit and promoting coordination and synergies with Member State activities. Dedicated support measures to assist the strategic approach and the process of priority setting are included in the ‘Inclusive, innovative and secure societies' challenge.

### Horizon 2020 basic documents

•COM/2011/0808 Horizon 2020—The Framework Programme for Research and Innovation—Communication from the Commission.

•COM/2011/0809 Proposal for a Regulation of the European Parliament and Council establishing Horizon 2020—the Framework Programme for Research and Innovation (2014–2020).

•COM/2011/0810 Proposal for a Regulation of the European Parliament and Council laying down the rules for the participation and dissemination in Horizon 2020.

•COM/2011/0811 Proposal for a Council Decision establishing the Specific Programme implementing Horizon 2020.

•COM/2011/0812 Proposal for a Council Regulation on the research and training programme of the European Atomic Energy Community (2014–2018) complementing Horizon 2020.

•COM/2011/0817 Proposal for a Regulation of the European Parliament and of the Council amending Regulation (EC) No 294/2008 establishing the European Institute of Innovation and Technology (EIT).

•COM/2011/0822 Proposal for a Decision of the European Parliament and of the Council on the Strategic Innovation Agenda of the European Institute of Innovation and Technology (EIT): the contribution of the EIT to a more innovative Europe.

## New European programme as the optimal instrument to promote the global leadership in medical innovation, excellent science and advanced health care

### Horizon 2020—Societal challenge *Health, demographic change and well-being*

‘…to improve the lifelong health and well-being of all.’

**Figure 14:**
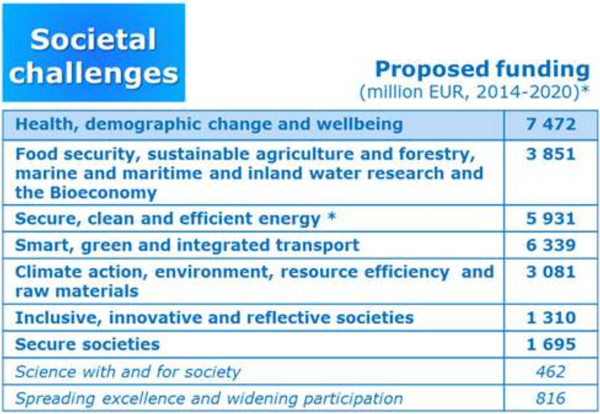
Societal challenges prioritised within the Horizon 2020.

Health, demographic change and well-being is the first and financially significantly supported societal challenge (see Figure 14).

Lifelong health and well-being for all, high-quality and economically sustainable health and care systems are the key words of Horizon 2020 and will make a major contribution to Europe 2020 strategy.

Europe will be for this 7-year period facing several challenges:

•Great demographic change: the number of Europeans aged over 65 is expected to nearly double from 85 million in 2008 to 151 million by 2060, and those over 80 to rise from 22 to 61 million in the same period.

•Chronic diseases such as cardiovascular disease (CVD), cancer, diabetes, neurological and mental health disorders, overweight and obesity and various functional limitations are major health and societal problems for the future. In Europe, CVD annually accounts for more than 2 million deaths and costs the economy more than EUR 192 billion while cancer accounts for a quarter of all deaths and is the number one cause of death in people aged 45–64. Over 27 million people in the EU suffer from diabetes and the total cost of brain disorders (including, but not limited to those affecting mental health) has been estimated at EUR 800 billion.

•The research and development of new drugs and vaccines are becoming more expensive and less effective.

•Health and illnesses cannot be solved at national levels separately; the coordinated European approach will enhance to eliminate fragmentation and duplicity in research, will contribute to addressing these challenges and deliver better health and well-being for all European citizens.

•The innovative approach depends on excellence in research to improve our fundamental understanding of health, disease, disability, development and ageing (including of life expectancy), and on the seamless and widespread translation of the resulting and existing knowledge into innovative and effective products, strategies, interventions and services.

•As a new progressive feature is the implementation of ICT techniques, technologies and tools. These include the development of long-term cohorts and the conduct of clinical trials, the clinical use of ‘omics’ or the development of ICT and their applications in health care practice, notably eHealth and mHealth.

•Big data means that biomedical research and health care bring every minute new information, data/data sets about an individual, cohort, population. Big data is difficult to collect, store, save, share and work with.

•The requirements of specific populations are also best addressed in an integrated manner, in the development of stratified and/or personalised medicine, in the treatment of rare diseases, and in providing assisted and independent living solutions.

•Coordinated European effort will contribute to the ongoing development of the European Research Area (ERA). It will interface with activities developed in the context of the Health for Growth Programme and the European Innovation Partnership on Active and Health Ageing.

### Philosophy of health care is changing

Health care is changing

•From illness to health.

•From treatment to prevention and early diagnostics.

•From approach one-fits-all to personalised medicine.

Effective health promotion prevents disease, improves well-being and is cost effective. Health promotion and disease prevention depend on an understanding of the determinants of health, on effective preventive tools, such as vaccines, on effective health and disease surveillance and preparedness, and on effective screening programmes.

An increasing disease and disability especially in an ageing population places further demands on health and care sectors. If effective health and care is to be maintained for all ages, efforts are required to improve decision-making in prevention and treatment provision, to identify and support the dissemination of best practice in the health and care sectors and to support integrated care and the wide uptake of technological, organisational and social innovations empowering, in particular, older persons as well as disabled persons to remain active and independent. Doing so will contribute to increasing and lengthening the duration of their physical, social and mental well-being.

## Horizon 2020 provides unlimited room for research and implementation in Predictive, Preventive and Personalised Medicine

### Integrated, sustainable, citizen-centred care (*this topic is considered within Horizon 2020*)

**Figure 15:**
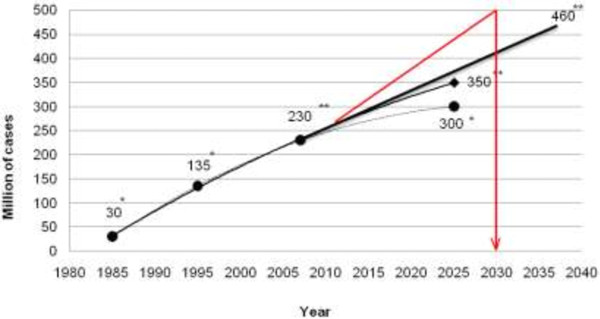
**Worldwide prognosis of the DM pandemic.** *Estimations as published around the year 2000. **Stepwise worsening prognosis as published in 2003–2008. Current prognosis is marked in *red colour*[10].

As a consequence of the accumulating clinical data and knowledge about the epidemiology and pathological mechanisms of the most frequent causes of morbidity and mortality, we are currently reconsidering our view of the origins and progression of cardiovascular, oncologic and neurodegenerative diseases. The majority of these pathologies are of chronic nature: they progress from precursor lesions over one or even several decades of life; therefore, it is often too late for effective therapeutic intervention. An excellent example is the pandemic of type 2 diabetes mellitus witnessed in the European Union. In most industrialised countries and countries with large populations, the permanently growing cohort of diabetics creates a serious health care problem and a dramatic health economic burden. The estimate for diabetes prevalence in the years 2025–2030 is half a billion patients worldwide (see Figure 15).

**Figure 16:**
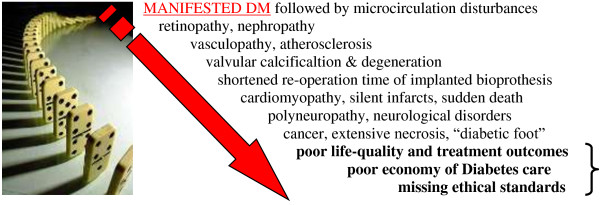
**Severe complications/comorbidities developed secondary to diabetes.** From ‘upstream’ (*up*) to ‘downstream’ (*bottom*) in the cascade of pathologic processes [10].

Moreover, the contemporary onset of the dominant type 2 diabetes was already observed in subpopulations of teenagers. Severe complications secondary to early onset of diabetes mellitus, such as retinopathy, nephropathy, silent ischaemia, dementia and cancer (Figure 16), soon may lead to collapsing health care systems.

**Figure 17:**
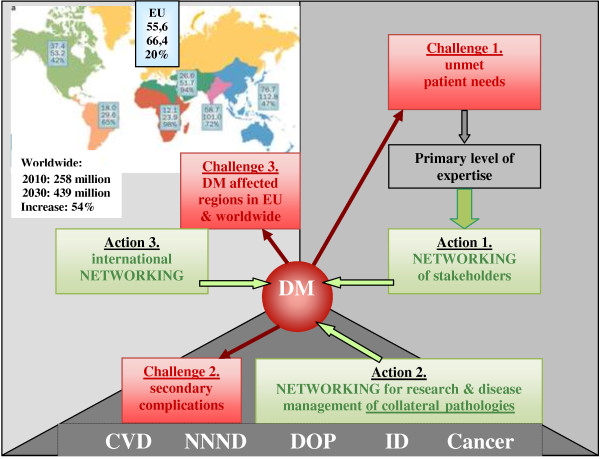
**PPPM in pre/diabetes care.** 3-Dimensional trans-domain coordinating and networking action by the EPMA responding to the challenges 1, 2 and 3, respectively. *CVD* cardiovascular disease, *NNND* neurological, neuropsychiatric and neurodegenerative diseases, *DOP* dental and oral pathologies, *ID* infectious diseases. *DM affected regions*: DM prevalence (million of patients) recorded in 2010 and predicted for 2030 with corresponding percentage of increase.

Clearly, the multidimensional features of diabetes mellitus (DM) require a systematic approach, with the best coordinated involvement of collaborative, multidisciplinary inter/national teams of clinicians, scientists and health care professionals across the spectrum of the medical field. The *3-dimensional trans-disciplinary coordinating and networking action* across the professional fields might be the best instrument within the Horizon 2020 in order to respond to the listed challenges as schematically demonstrated in Figure 17. This integrative well-coordinated medical approach utilises the innovation by predictive diagnostics as the basis for concomitant targeted prevention, patient profiling as the basis for individualised treatment algorithms and medical approaches tailored to the patient. Creation of innovative ‘proof-of-principle’ for health and disease management followed by new guidelines in health care should create a robust juristic and economic platform for advanced medical services utilising the cost-effective models of risk assessment followed by tailored treatments focussed on the precursor stages of non-communicable chronic disease [11].

### Understanding health, ageing and disease (*this topic is considered within Horizon 2020*)

**Figure 18:**
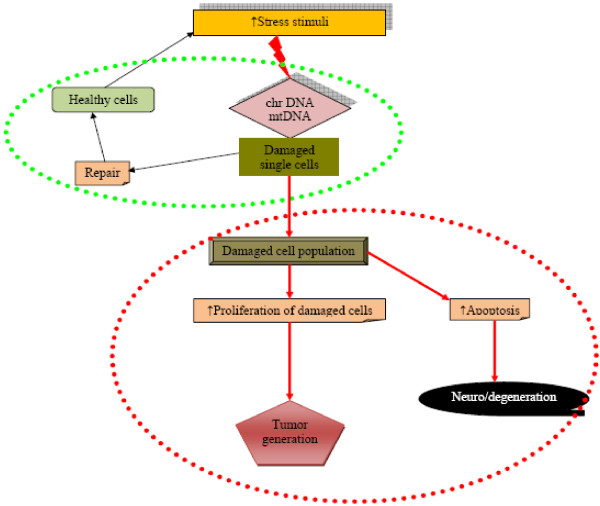
**Potential mechanisms for the increased risk of both degenerative processes and malignancies under stress condition.** The first part circled in *green colour* shows physiologic repair of damaged single cells. The second part circled in *red colour* demonstrates pathophysiologic processes leading either to degenerative or malignant transformations [12].

A number of molecular mechanisms have received considerable attention in contributing to the understanding of the pathogenesis underlying DM and its attendant complications (see Figure 18). However, the complex and intrusive nature of the disease means that the quest (involving diverse scientific domains and disciplines) must continue to better unravel contributing mechanisms of initiation and progression of DM and its target organ complications. This multidisciplinary paradigm is essential to bridge the substantial gap between research and clinical sectors, in a translational fashion. Identification of pathogenic mechanisms, therapeutic targets and biomarker sets to predict, prevent and personalise treatment of DM patients is the central focus of such an orchestration (Challenge/Action 1 ‘Networking of stakeholders’, see Figure 17). These concepts create a feasible and credible professional platform aimed at providing proof-of-principle models for advanced health and disease management.

### Innovative treatments and technologies (*this topic is considered within Horizon 2020*)

**Figure 19:**
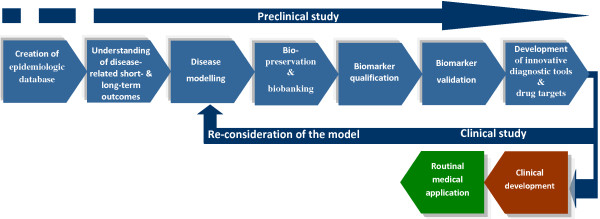
**Moving from basic research to clinical implementation.** Essential steps in creating the evidence-based platform for innovative technologies for early/predictive diagnostics and personalised treatments [13].

Optimal health care intervention is a cascade of events functionally linked to each other and based on the innovative technologies that allow for individual prediction, targeted prevention and treatments tailored to the person. This innovation, before becoming successfully applied in daily medical practice, should be carefully created at the level of preparatory measures and individual research steps moving from basic research to the clinical implementation as summarised in Figure 19. Without the adequate support of the excellent science and technologies within the nominated steps, a successful implementation of innovative treatments was rather illusive. This comprehensive support is the content of the long-lasting Horizon 2020.

### Improving diagnosis (*this topic is considered within Horizon 2020*)

**Figure 20:**
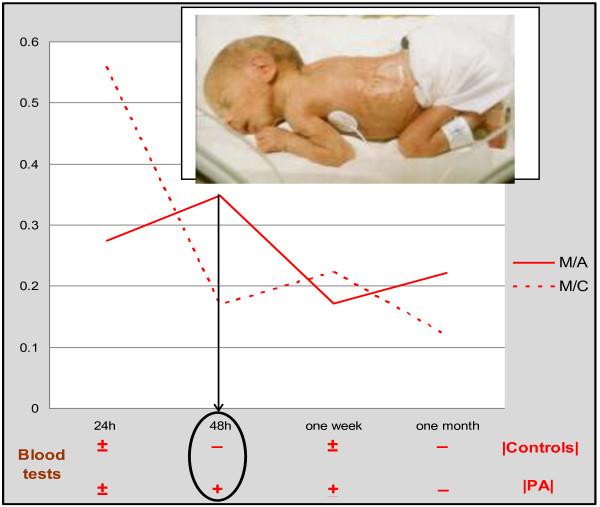
**Specialised screening programmes and targeted preventive measures.** Specialised screening programmes and targeted preventive measures are essential to be performed in newborns for timely protection against severe outcomes of perinatal complications such as birth asphyxia. *An example*: the correlation of TAU-protein levels (see the diagram) found between brain (mesencephalon, *M*) and blood samples and asphyxic newborns (*A*, *PA*) versus controls (*C*) allows the performance of minimally invasive blood tests to determine the specific brain damage potentially leading to sever long-term outcomes such as tauopathies (Alzheimer's disease and others) [14].

Perhaps the best example for this task represents the cohort of individuals affected by birth asphyxia. According to the currently available statistical data, a perinatal asphyxia (insufficient oxygen supply) is the most frequent birth complication with the prevalence between 1 and 100 cases per 1,000 live births in diverse populations worldwide (see Figure 20).

**Figure 21:**
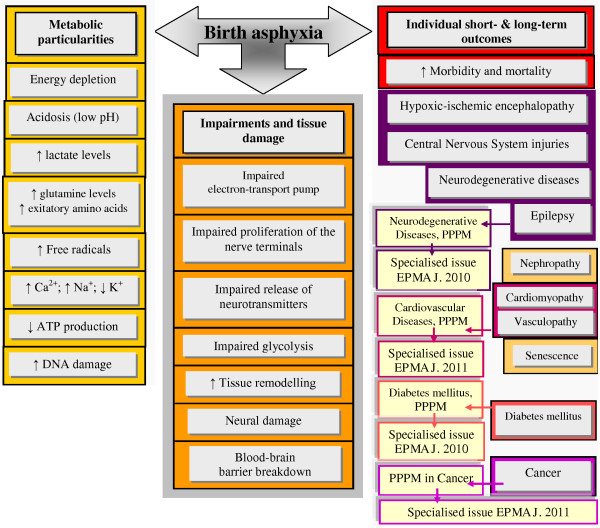
**Metabolic particularities, impairments and individual outcomes by perinatal asphyxia.***PPPM* predictive, preventive and personalised medicine. Adapted from [14].

Secondary to perinatal (birth) asphyxia, a postnatal manifestation of hypoxic-ischaemic encephalopathy (HIE) is frequently observed being associated with either mild or severe organ damage in asphyxiated newborns, both leading to the development of chronic pathologies. The severe insults often cause neurodegenerative diseases, mental retardation and epilepsies manifested later in life. The mild insults lead to so-called ‘minimal brain-damage disorders’ such as attention deficits and hyperactivity, but can also be associated with the development of schizophrenia, lifelong functional psychotic syndromes and damage and pathologies of other life-important organs (the kidney, heart, vascular system and others). In some particular cases, it is difficult to discriminate between mild and severe asphyxia: advanced methodology to improve diagnosis of birth asphyxia and prediction of individual short- and long-term outcomes obligatorily needs to be developed. The complete overview to the central biological processes affected by perinatal asphyxia is schematically presented in Figure 21. The figure provides comprehensive information about the metabolic particularities of the hypoxic processes, potential impairments and short- versus long-term outcomes which can be expected with individually manifested grade of severity. Hypoxic-ischaemic encephalopathy, CNS damage, epilepsy, nephropathy, cardiomyopathy, vascular pathologies, senescence, diabetes mellitus, cancer, neurodegenerative diseases, morbidity and mortality and tissue remodelling all belong to individual short- and long-term outcomes of birth asphyxia [14]. Specialised issues of *The EPMA Journal* have been dedicated to the ‘PPPM in Diabetes’, ‘PPPM in Neurodegenerative Diseases’, ‘PPPM in Cancer’, ‘PPPM in Cardiovascular Diseases’, ‘PPPM in Body Culture and Sport Medicine’, ‘PPPM in Health care Overview’, where the articles overview the innovative technologies of predictive diagnostics, targeted prevention measures, desirable personalisation of medical care, individual prediction, targeted prevention and personalised treatments before a manifestation of lifelong chronic pathologies.

### Effective health promotion, disease prevention, preparedness and screening (*this topic is considered within Horizon 2020*)

**Figure 22:**
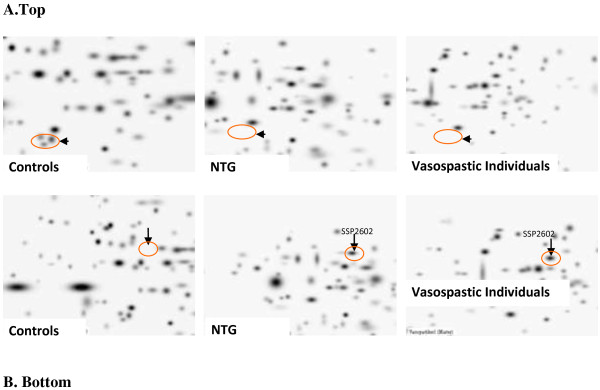
**Proteomics imaging of blood biomarkers (*ex vivo *identification in circulating leukocytes) specific for normal-tension glaucoma (NTG). (A)***Top*: The pathology-specific protein-cluster is completely suppressed in both NTG and vasospasm in contrast to controls. **(B)***Bottom*: The marked protein SSP2602 is highly up-regulated in both NTG and vasospasm; this protein normally does not express by circulating leukocytes of controls—healthy individuals without vasospasm [18].

Paradigm change from delayed reactive medicine to predictive diagnostics followed by targeted prevention before manifestation of pathology presents innovative concept for advanced health care. Pre-selection of healthy but pathology-predisposed individuals represents the primary task in the overall action. One of the best examples might be given on the cohort of healthy vasospastic individuals who, however, represent a group of risk strongly predisposed to several pathologies due to vascular dysregulation with concomitantly shifted gene expression and other syndrome-specific alterations at molecular and subcellular levels as it is demonstrated in Figure 22. Since vasospastic syndrome is a frequent phenomenon in adolescence and young adults, this makes the task of prediction and targeted prevention of downstream-related pathologies particularly attractive from several points of view including economical aspects [15-17]. Protein patterns in circulating leukocytes demonstrate clear similarities between vasospasm and normal-tension glaucoma versus controls. Moreover, protein clusters can be considered to develop the biomarker sets for predictive imaging of healthy vasospastic individuals predisposed to glaucoma as shown in Figure 22. Subcellular imaging and ‘gene hunting’ investigations provide further evidence for vasospasm as predisposition to glaucoma. However, development of some other related pathologies cannot be excluded. Predictive molecular profiling in the blood can specify individual predisposition for effective prevention with low costs. Alternatively to *omics technologies, diagnostic and prognostic approaches utilising circulating nucleic acids in plasma and serum, urine, saliva, and tear fluid can be of great importance. New guidelines are essential to regulate the field of non-invasive predictive technologies to improve personalised treatment of healthy but pathology-predisposed groups of risk. The instruments offered by Horizon 2020 are essential to motivate both health care providers and patients for participative medicine (active cooperation between patient and physician) before manifested pathologies.

### Accelerated ageing or inadequate health care? Advancing active and healthy ageing with utilising instruments of Horizon 2020 (*this topic is considered within Horizon 2020*)

#### Current figures

Over 300 million diabetics frequently affected by poly-neuropathy as secondary complication, 18 million patients with Alzheimer's disease (also diagnosed as diabetes type 3), neurodegenerative eye diseases with leading causes of blindness—diabetic retinopathy and estimated 70 million glaucoma patients worldwide, millions of patients with Parkinson's disease, multiple sclerosis, epilepsy, cerebral palsy and dementia in the elderly—these are all the patient cohorts with severe neurological disorders, who demonstrate dramatically decreased life quality. Progressive neurodegeneration is the common feature of multifactorial physical and cognitive disability. Permanently increasing numbers of those patients as well as dramatic disease-related economical burden indicate that current education, research and health care in this field are far from optimal and should be corrected. Since neurodegeneration is a chronic process, which begins long before the onset of clinically diagnosed symptoms, currently there is an evident lack of targeted prevention in any age-category inchoate with early childhood, over adolescence until late adulthood [19].

#### Optimistic versus pessimistic prognosis

This depends much on diagnostic, preventive and treatment approaches which health care will preferably adopt in the near future. Without innovation in health care, neurodegenerative disorders can reach more than 30% of global disease burden until 2020. In contrast, effective utilisation of advanced early/predictive diagnostics, preventive and personalised medical approaches could enable a significant portion of population to reach the 100-year age limit remaining vibrant in excellent physical and mental health as actively contributing members of society. Specialised calls of Horizon 2020 aim at solving the accumulated problems and promote active and healthy ageing, early risk detection, mental well-being within ageing populations, application of ICT technologies for independent living with cognitive impairments as well as robotics services within assisted living environments.

### Improving health information, data exploitation and providing an evidence base for health policies and regulation (*this topic is considered within Horizon 2020*)

Through the support of the Alexander von Humboldt Foundation in the years 2012–2013, EPMA has performed a specialised project focused on the collaboration among PPPM-related professional groups and policymakers. This project was aimed at identifying problems and deficits in health care of common and pandemic chronic diseases such as type 2 diabetes mellitus. Interviews performed with experts from all countries of the European Union, Israel, Russia and Turkey resulted in the conclusion that one of the common deficits is a missing communication and collaboration between individual professional groups on one side and health care professionals and decision-makers on the other side. Moreover, there are difficulties in accessing national regulatory bodies to enhance the message of patient-focused health care. This evidence demonstrates that decisions in the health care sector are generally made without consideration of accumulated professional knowledge. This may explain the lack of sufficient efficacy currently observed in health care systems.

In contrast, if well established, the collaboration with governmental bodies may lead to the reconsideration of current national and European guidelines for health care-relevant research activities and medical services both advanced by PPPM. There are following emerging issues to be within the instruments and programmes of Horizon 2020:

•Promoting research fields focused on the patient needs

•Mandating a paradigm shift from reactive to predictive and preventive medicine.

•Creating specialised state budgets to target preventive measures against pandemic incidence of non-communicable chronic diseases.

•Forcing an effective implementation of innovative predictive diagnostics.

•Advancing bioinformatics to meet biomedical and clinical needs.

•Facilitating digital representation of health data to improve professional communication and optimise diagnostic and treatment approaches.

•Introducing optimal medical records.

•Standardising predictive technologies for human safety testing.

•Creating new educational programmes for professionals to implement integrative medical services in predictive, preventive and personalised medicine.

•Creating educational measures to increase the understanding of innovative diagnostic technologies and targeted preventive measures in the general population.

•Creating new economic models to motivate health responsibility.

As health care is subject to national subsidiarity, there are substantial variations in the level of provision in different countries. However, corresponding standards might be introduced at the European level to promote inter-European mobility and to avoid economic discrepancies and conflict of interest [20].

### Comments on and ‘ready-to-go’ information about the first calls released for 2014–2015

The most important features of SC1 especially for the two open calls 2014, 2015 are the following: improving health promotion and disease prevention; understanding disease and improving diagnosis; developing effective screening programmes and improving the assessment of disease susceptibility; improving surveillance and preparedness; developing better preventive vaccines; using *in silico* medicine for improving disease management and prediction; treating disease; transferring knowledge to clinical practice and scalable innovation actions; better use of health data; active ageing, independent and assisted living; individual empowerment for self-management of health; promotion of integrated care; improving scientific tools and methods to support policy making and regulatory needs; optimising the efficiency and effectiveness of health care systems and reducing inequalities by evidence-based decision-making and dissemination of best practice; and innovative technologies and approaches. Work programme for 2014 and 2015 [21] (see also Tables 1 and 2) consists of seven thematic areas with 34 topics for project proposals, ten coordination actions and other activities:

1. Understanding health, ageing and disease.

PHC 1–2014: Understanding health, ageing and disease: determinants, risk factors and pathways.

PHC 2–2015: Understanding diseases: systems medicine.

PHC 3–2015: Understanding common mechanisms of diseases and their relevance in comorbidities.

2. Effective health promotion, disease prevention, preparedness and screening.

PHC 4–2015: Health promotion and disease prevention: improved intersector cooperation for environment- and health-based interventions.

PHC 5–2014: Health promotion and disease prevention: translating omics into stratified approaches.

PHC 6–2014: Evaluating existing screening and prevention programmes.

PHC 7–2014: Improving the control of infectious epidemics and foodborne outbreaks through rapid identification of pathogens (see also societal Challenge 2).

PHC 8–2014: Vaccine development for poverty-related and neglected infectious diseases: tuberculosis.

PHC 9–2015: Vaccine development for poverty-related and neglected infectious diseases: HIV/AIDS.

3. Improving diagnosis.

PHC 10–2014: Development of new diagnostic tools and technologies: *in vitro* devices, assays and platforms.

PHC 11–2015: Development of new diagnostic tools and technologies: *in vivo* medical imaging technologies.

PHC 12–2014/2015: Clinical research for the validation of biomarkers and/or diagnostic medical devices.

4. Innovative treatments and technologies.

PHC 13–2014: New therapies for chronic non-communicable diseases.

PHC 14–2015: New therapies for rare diseases.

PHC 15–2014/2015: Clinical research on regenerative medicine.

PHC 16–2015: Tools and technologies for advanced therapies.

PHC 17–2014: Comparing the effectiveness of existing health care interventions in the elderly.

PHC 18–2015: Establishing effectiveness of health care interventions in the paediatric population.

5. Advancing active and healthy ageing.

PHC 19–2014: Advancing active and healthy ageing with ICT: service robotics within assisted living environments.

PHC 20–2014: Advancing active and healthy ageing with ICT: ICT solutions for independent living with cognitive impairment.

PHC 21–2015: Advancing active and healthy ageing with ICT: early risk detection and intervention.

6. Integrated, sustainable, citizen-centred care.

PHC 23–2014: Developing and comparing new models for safe and efficient, prevention oriented health and care systems:

PHC 24–2015: Piloting personalised medicine in health and care systems.

PHC 25–2015: Advanced ICT systems and services for Integrated Care.

PHC 26–2014: Self-management of health and disease: citizen engagement and mHealth.

PHC 27–2015: Self-management of health and disease and patient empowerment supported by ICT.

PHC 28–2015: Self-management of health and disease and decision support systems based on predictive computer modelling used by the patient him or herself.

PHC 29–2015: Public procurement of innovative eHealth services.

7. Improving health information, data exploitation and providing an evidence base for health policies and regulation.

PHC 30–2015: Digital representation of health data to improve disease diagnosis and treatment.

PHC 31–2014: Foresight for health policy development and regulation.

PHC 32–2014: Advancing bioinformatics to meet biomedical and clinical needs.

PHC 33–2015: New approaches to improve predictive human safety testing.

PHC 34–2014: eHealth interoperability.

**Table 1:** **Information about the calls released for thematic areas in 2014–2015: schedule, taken from**[21]

**Theme**	**Stage/phase**		**Stage/phase**	
PHC 1–2014	Stage 1—11th March 2014 at 1700 hours, Brussels time		Stage 2—19th August 2014 at 1700 hours, Brussels time	
PHC 5–2014				
PHC 6–2014				
PHC 10–2014				
PHC 13–2014				
PHC 17–2014				
PHC 23–2014				
PHC 32–2014				
PHC 7–2014	Single stage—15th April 2014 at 1700 hours Brussels time			
PHC 8–2014				
PHC 15–2014/2015				
PHC 19–2014				
PHC 20–2014				
PHC 26–2014				
PHC 31–2014				
PHC 34–2014				
PHC 12–2014/2015	Phase 1	Phase 2	Phase 1	Phase 2
	18th June 2014	09th October 2014	{18th March 2015 17th June 2015 17th September 2015 16th December 2015}	{18th March 2015 17th June 2015 17th September 2015 16th December 2015}
Open call cut-off dates	24th September 2014	17th December 2014		
Open from 1st March 2014 for phase 1 and phase 2^a^	17th December 2014			

Conditions for this call: Publication date 11 December 2013. Deadline(s) (2014): 11 March 2014 (stage 1 of two stage calls), 15 April 2014 (single stage call), 19 August 2014 (stage 2 of two stage calls), PHC 12–2014/2015 (as below). The Director General responsible may delay this deadline by up to 2 months. Any deadlines provided in brackets are indicative and are subject to a separate financing decision for 2015. ^a^The Director General responsible may delay this date by up to 2 months.

**Table 2:** **Information about calls released for thematic areas in 2014–2015: indicative budget for calls, taken from**[21]

**Theme**	**2014**	**2015**	**Stage**
	**EUR million**	**EUR million**	
PHC 1–2014	54.00		Two stage
PHC 2–2015		36.00	Two stage
PHC 3–2015		30.00	Two stage
PHC 4–2015		18.00	Two stage
PHC 5–2014	24.00		Two stage
PHC 6–2014	15.00		Two stage
PHC 7–2014	15.00 (with an additional 5.00 million from SC2)		Single stage and hearing
PHC 8–2014	25.00		Single stage and hearing
PHC 9–2015		21.00	Single stage and hearing
PHC 10–2014	48.00		Two stage
PHC 11–2015		47.00	Two stage
PHC 12–2014/2015	66.10, out of which 6.61 for phase 1, 58.17 for phase 2 and 1.32 for mentoring and coaching support and phase 3	45.00, out of which 4.50 for phase 1, 39.60 for phase 2 and 0.90 for mentoring and coaching support and phase 3	SME instrument
PHC 13–2014	60.00		Two stage
PHC 14–2015		60.00	Two stage
PHC 15–2014/2015	36.00	35.00	2014–one stage, one deadline
PHC 16–2015		36.00	Two stage
PHC 17–2014	48.00		Two stage
PHC 18–2015		26.00	Two stage
PHC 19–2014	24.60		Single stage
PHC 20–2014	10.00		Single stage
PHC 21–2015		21.00	Single stage
PHC 22–2015		17.00	Two stage
PHC 23–2014	30.00		Two stage
PHC 24–2015		30.00	Two stage
PHC 25–2015		20.00	Single stage
PHC 26–2014	59.60		Single stage
PHC 27–2015		15.00	Single stage
PHC 28–2015		20.00	Single stage
PHC 29–2015		10.00	Single stage
PHC 30–2015		20.00	Single stage
PHC 31–2014	6.00		Single stage
PHC 32–2014	24.00		Two stage
PHC 33–2015		30.00	Two stage
PHC 34–2014	4.00		Single stage
TOTAL	549.30	537.00	

Indicative budget: EUR 549.30 million from the 2014 budget, of which EUR 5.00 million from the societal challenge ‘Food security, sustainable agriculture and forestry, marine and maritime and inland water research and the bioeconomy’ and subject to the availability of the appropriations provided for in the draft budget for 2014 after the adoption of the budget for 2014 by the budgetary authority or if the budget is not adopted as provided for in the system of provisional twelfths. EUR 537.00 million from the 2015 budget (the budget amounts are indicative and will be subject to a separate financing decision to cover the amounts to be allocated for 2015). Single stage for both phase 1 and phase 2. The budget available for phase 1 and phase 2 will be divided equally between each cut-off date.

Coordination activities summarised in Tables 3 and 4 are the following:

HCO 1–2014: Support for the European Innovation Partnership on Active and Healthy Ageing.

HCO 2–2014: Joint Programming: Coordination Action for the Joint Programming Initiative (JPI) ‘More Years, Better Lives—the Challenges and Opportunities of Demographic Change’.

HCO 3–2015: Support for the European Reference Networks: efficient network modelling and validation.

HCO 4–2014: Support for international infectious disease preparedness research.

HCO 5–2014: Global Alliance for Chronic Diseases: prevention and treatment of type 2 diabetes.

HCO 6–2015: Global Alliance for Chronic Diseases: 2015 priority.

HCO 7–2014: ERA-NET: Establishing synergies between the Joint Programming on Neurodegenerative Diseases Research and Horizon 2020.

HCO 8–2014: ERA-NET: Aligning national/regional translational cancer research programmes and activities.

HCO 9–2014: ERA-NET: Systems medicine to address clinical needs.

HCO 10–2014: ERA NET: Rare disease research implementing IRDiRC objectives.

HCO 11–2015: ERA-NET: Collaboration and alignment of national programmes and activities in the area of brain-related diseases and disorders of the nervous system.

HCO 12–2015: ERA-NET: Antimicrobial resistance.

HCO 13–2015: ERA-NET: Cardiovascular disease.

HCO 14–2014: Bridging the divide in European health research and innovation.

HCO 15–2014: Mobilisation and mutual learning action plan.

HCO 16–2014: National Contact Points.

**Table 3:** **Full information about the calls dedicated to the coordination measures, taken from**[21]

	**Schedule**
HCO 1–2014	15 Apr. 2014 at 1700 hours Brussels time
HCO 2–2014	
HCO 4–2014	
HCO 5–2014	
HCO 7–2014	
HCO 8–2014	
HCO 9–2014	
HCO 10–2014	
HCO 14–2014	
HCO 15–2014	
HCO 16–2014	

Conditions for this call: Publication date December 11, 2013. Deadline, 15 April 2014. The Director General responsible may delay this deadline by up to 2 months.

**Table 4:** **Indicative budget, taken from**[21]

	**2014**	***2015***	**Stage**
	**EUR million**	***EUR million***	
HCO 1–2014	2.00		Single stage
HCO 2–2014	2.00		Single stage
HCO 3–2015		*2.00*	Single stage
HCO 4–2015	3.00		Single stage
HCO 5–2014	9.00		Single stage
HCO 6–2015		*12.00*	Single stage
HCO 7–2014	5.00		Single stage
HCO 8–2014	5.00		Single stage
HCO 9–2014	5.00		Single stage
HCO 10–2014	5.00		Single stage
HCO 11–2015		*5.00*	Single stage
HCO 12–2015		*5.00*	Single stage
HCO 13–2015		*5.00*	Single stage
HCO 14–2014	1.00		Single stage
HCO 15–2014	1.00		Single stage
HCO 16–2014	2.00		Single stage
TOTAL	40.00	*29.00*	

The EUR 40.00 million from the 2014 budget is subject to the availability of the appropriations provided for in the draft budget for 2014 after the adoption by the budgetary authority or if the budget is not adopted as provided for in the system of provisional twelfths. EUR 29.00 million from the 2015 budget amounts is indicative and will be subject to a separate financing decision to cover the amounts to be allocated for 2015.

Other actions are also available:

HOA 1–2014/2015: Subscription fee: Human Frontier Science Programme Organisation.

HOA 2–2014/2015: Tenders for programme evaluation, studies and impact assessment and for conferences, events and outreach activities.

HOA 3–2014/2015: Presidency events—eHealth.

HOA 4–2014/15: Independent experts assisting in proposal evaluations and project reviews.

HOA 5–2014: Grant to the Global Alliance for Chronic Diseases.

HOA 6–2014: Stem cell research outreach.

HOA 7–2015: eHealth Sectoral Inducement Prize.

HOA 8–2015: Inducement prize.

For getting all relevant information about the upcoming call in Horizon 2020 SC1, every participant has to register at PARTICIPANT PORTAL [22] then in main menu go to FUNDING OPPORTUNITIES, in the central column find SOCIETAL CHALLENGES and click on HEALTH, DEMOGRAPHIC CHANGE AND WELLBEING, and finally the newly opened screen is the page with all information about the topics and for project proposal submission [21].

## EPMA strategies within Horizon 2020

### Health care paradigm shift from delayed reactive to Predictive, Preventive and Personalised Medicine: innovation, scientific and technological excellence

**Figure 23:**
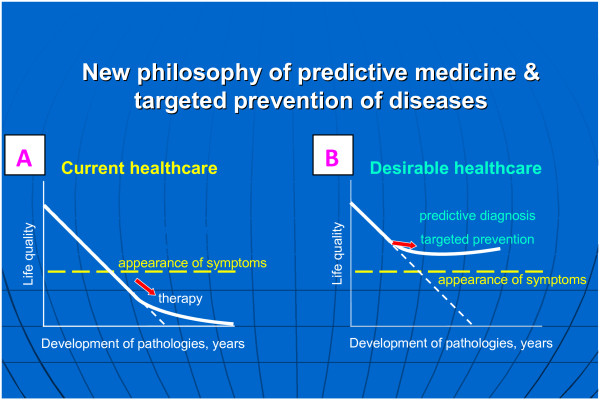
**Health care approaches.** Current health care approach **(A)** versus advanced health care approach **(B)**. The advanced health care approach considers a paradigm change from delayed interventional to predictive, preventive and personalised medicine as the robust platform for optimal medical services; figure is taken from [23].

For many acute and chronic disorders, the current health care outcomes are considered as being inadequate [1,20]. Current health care practices essentially rely on the emergence of signs and symptoms of human pathologies prior to initiation of interventional modalities as illustrated in Figure 23A. Consequently, long-term morbidity and prognosis may often be poor but the associated costs are extremely high and permanently increasing year-by-year worldwide. Most impressive examples include the pandemic of type 2 diabetes mellitus, neurodegenerative disorders and some types of cancer, which, over the next 10–20 years, associated with the economic situation, could spell disaster for health care systems on a global scale. A desirable strategy is illustrated in Figure 23B. The major premise of the advanced health care approach is the paradigm change from reactive to predictive medicine, from delayed to preventive and personalised medicine. This requires the application of innovative biotechnologies to predict human pathologies, the devising of appropriate and timely preventive strategies and individualised treatment planning.

### Predictive and prognostic tests: why and how to realise in medical practice

The following actualities motivate the introduction of predictive and prognostic approaches into daily medical services:

–Reactive disease care.

–Delayed intervention.

–Untargeted medication.

–Overdosed and poisoned patients.

–Low effectiveness of treatments.

Current deficits in medical services such as missing toxicological examination of the prescribed medication and ethically problematic delayed reactive and inadequate treatments require new strategies for a targeted prevention of diseases and negative side effects of treatments [24]. Clinically validated predictive and prognostic tests provide new perspectives on how to advance and personalise medical services.

Accuracy, sensitivity and specificity of predictive and prognostic tests are the premise of advanced health care. There is the evident necessity to enable globally comparable investigative, diagnostic and prognostic strategies to be reliably implemented. This requires regulatory oversight and meaningful engagement with *in vitro* diagnostic providers. The globalisation of markets and laboratory-related business requires the comparability, or the unification, of laboratory test values. The mobility of both patients and health care professionals as well as the increasing global data flow require such comparability [25]. Hence, the unification of laboratory tests should be placed at the top of the list of corresponding adapting measures.

In order to create a robust investigative platform, the utilisation of appropriate predictive and prognostic approaches should be mandated, which means an extensive collaboration between relevant professional organisations on one side and policymakers on the other side resulting in a well-organised promotion and widespread application of the predictive medical care in health care systems.

### Multidisciplinary analytical expertise is essential for optimal clinical decisions

Optimal clinical decisions result from a multidisciplinary approach performed by specialists with complementary analytical expertise. Therefore, the overall task is formulated as the integrative medical approach of the multimodal diagnostics, application of health- versus disease-specific biomarker patterns, creation of individual patient profiles and widespread medical records which altogether allow for treatments tailored to the person [13]. Multimodal diagnostics (i.e. deep, early and predictive diagnostics) represents a model-based procedure with several levels of examination resulting in extended patient profiles and medical records which obligate inclusion of an interview with the patient, application of multidisciplinary questionnaires, medical imaging, *in vitro* diagnostics and accurate evaluation of pathology-relevant and potential risk factors. For laboratory diagnostics, it is highly recommended to perform analysis at complementary levels and consider stage-specific molecular patterns, such as DNA polymorphisms, transcriptional regulation, shifted protein expression patterns, post-translational modification, stage-specific subcellular imaging and altered activity of enzymatic complexes.

### Consequent paradigm change in health care is the reconsideration of competencies and responsibilities in the laboratory-clinician interface

Shifting the role of laboratory medicine from the ‘passive performing’ to the ‘active advising’ is the consequent paradigm change in health care resulting from the above-listed necessities and expected innovations. This reconsideration of the laboratory-clinician interface might significantly advance the quality of current medical services, although the implementation of this approach across countries should be adapted to local conditions. Well-known motivating actualities for the reconsideration are, for example, so-called within-individual variations as well as grey-zone results, when individual laboratory parameters range between ‘healthy’ and ‘unhealthy’ zones and any interpretation is problematic for clinicians. Therefore, recommendations by the laboratory tests/interpretations to assist clinical practice are essential [20,26]. This assistance ranges from advising on the necessity for additional tests to the consequent analysis of the fluctuating targets. Additional tests should be considered from the viewpoint of their reasonability, in order to reach an accurate and realistic health-related data interpretation for the individual. The analysis of dynamic changes of the target is essential to evaluate potential health impacts such as an individual predisposition to the disease and/or a predictive diagnosis before a clinical manifestation of symptoms. Laboratory value-added investigation and data interpretation should be obligatory promoted to advance functional relationship between laboratory medicine and clinicians both as the responsible decision-makers [20,27].

### Healthy ageing and solutions for independent living with the long-term health impairments: demographic change and societal challenges

Current trends in Europe demonstrate dramatic demographic shifts in favour of elderly subpopulations. With type 2 diabetes mellitus, pandemic and its severe secondary complication such as stroke, 50% of people after 85 years of age are affected by Alzheimer's disease (also diagnosed as type 3 diabetes), neurodegenerative eye diseases which are the leading causes of blindness, diabetic retinopathy and estimated 70 million glaucoma patients worldwide, and there are millions with Parkinson's disease, multiple sclerosis, epilepsy and dementia in the elderly [28]. Optimistic versus pessimistic prognosis depends much on diagnostic, preventive and treatment approaches which health care systems will preferably adopt in the near future. Without innovation in health care, neurodegenerative disorders can reach more than 30% of the global disease burden until 2020. In contrast, effective utilisation of advanced early/predictive diagnostics, preventive and personalised medical approaches could enable a significant portion of population to reach the 100-year age limit remaining vibrant in excellent physical and mental health as actively contributing members of society. These realities require new strategies in medical services focused on maintaining physical and mental health of ageing populations. Further, to satisfy the needs of long-term monitoring of chronically diseased patients with a potential duration of some years to several decades of life, a new generation of point-of-care monitoring devices is required. These mobile health technologies must enable self-monitoring, distanced monitoring and the remote management of diseases of people for independent living with health impairments.

### Progressing from a ‘disease care’ to ‘health care’: ‘well-being’ concepts

The evident pandemic of common chronic diseases, the consequences of a reactive approach and hence poor economy of disease care as well as dramatic social and ethical problems that result from delayed medical services necessitate experts to reconsider the current paradigm of reactive disease care in favour of health care. This means that there is a mandated progressive implementation of a new philosophy of the well-being concept tailored to the person (age, gender, socio-economic status, individual predispositions, patient-specific profiles, etc.) as a spectrum of personalised measures for professional care that promotes the mental and physical health of an individual [24]. Consequently, better individual outcomes are expected with respect to less morbidity, better quality of life and improvement of both health economy and ethical standards [20,29,30].

### Forcing the practical application of health- versus disease-specific biomarker patterns: industrial leadership

Current calculations demonstrate a long-term trend of the dramatic crisis in the creation of clinically useful molecular entities (biomarkers, drug targets, etc.). Further, an innovation in *in vitro* diagnostic testing is hampered by a lack of funding for studies aiming at the effective validation of the clinical utility of novel biomarkers. Consequently, a deep analysis of how to shorten the time span between a discovery of new molecular targets, creation of valid health- versus disease-specific biomarker patterns and their effective utilisation in daily practice is the strategic point of highest priority [31]. Up to this point, expert recommendations have been summarised in the EPMA White Paper in 2012 [5]. The proposed measures include the following:

–Emerging technologies in population screening (faster, more precise, cheaper).

–Set-up of pathology-specific biomarker patterns instead of ineffective single molecules.

–Non- or minimally invasive diagnostic tools (saliva, urea, tear fluid, blood samples).

–Effective promotion of validation studies.

–Transparent schemes for functional contacts between researchers who discovered new targets and implementing industrial partners.

–Dramatic improvements in national and international patenting systems.

An effective implementation of the above-listed measures may favour more flexible small- and middle-sized industrial companies in the competitive process of PPPM-related innovation.

### Optimal organisation of biopreservation and biobanking

Well-organised biopreservation and biobanking is expected to create a robust platform for the optimal set-up of health- and disease-specific biomarker patterns and drug targets. Therefore, ethically correct and technologically excellent biopreservation and biobanking are central activities in the field of PPPM. As it has been formulated in the EPMA White Paper in 2012 [5], a list of essential requirements for the optimal organisation of biopreservation and biobanking should be functionally satisfied. Competencies should be harmonised among the main stakeholders, namely patients, scientific community and health care providers which includes commercial companies interested in *in vitro* diagnostics, development of biomarker patterns and novel drug targets. However, currently, biobanking is facing major viability challenges. As for individual types of biological material (tissue samples, blood samples, DNA, RNA, proteins, metabolites, etc.), national laws vary for local instructions and legal approaches and how samples may be collected, stored, retrieved and tested. In plenty of cases, this may be quite restrictive and incompatible with rules that are legal for other countries. The analytical quality of collections is frequently compromised as the storage condition for the samples is changing. Donation of samples to a biobank not only requires anonymity but also strict control over record linkage and access. Sample collections with a permission of specialised ethical commission should be hosted by viable academic units and health facilities with a proven record of research and should not be exploited for commercial gain but for the common good. Disease-focused collections require acquired samples to be retrospectively valid for a development of novel biomarkers. Such biomarkers need to have a demonstrated clinical utility prior to their introduction in medical services. For a disease-specific biobanking, the disease needs to have been well characterised for each patient, with immaculate record-keeping, to enable to draw conclusions on the new markers. These critical problems may be optimally solved by relevant professional groups with complementary expertise such as the European Federation of Clinical Chemistry and Laboratory Medicine, EPMA and ESBB followed by adequate decisions of policymakers resulting in the creation of a robust juristic platform. An international biobanking, if designed as being reliable for advanced health care services, needs an adequate juristic platform that considers the following interests of all parties involved in the research, business and health care, namely: (1) donors of biological materials, (2) clinicians providing patient records, (3) laboratory units collecting the samples, (4) storage units, (5) research analysing the collections and creating new diagnostic and drug targets, (6) patenting institutions, (7) health care industry, (8) patient cohorts, (9) groups at risk, (10) general population and (11) educators.

### Medical (patient) records as the central task for integrative bioinformatics

How can integrative analytical data in the above-delineated context and for the listed purposes be treated? A creation of innovative medical records is considered as a priority for scientific, coordinated and implementation-oriented programmes of multidisciplinary character. Further, creation of innovative medical records might be a strong driver for standardising communications in the broader health-related scientific community and health care industry [4,20,32]. However, with the exponential growth of published descriptive knowledge, on one hand, and the deluge of high-throughput data generated by advanced technologies, on the other hand, there is a strong need for strategies to manage, analyse and interpret the huge amounts of data generated by different research disciplines and clinical fields relevant for predictive diagnostics, targeted prevention and treatments tailored to the person. Hence, for successful translation of research findings into clinical applications beneficial for patients, the data and knowledge should be accessible to multiple research disciplines that collaborate together on a particular health-related topic. For that, the generated data and knowledge should be appropriately collected, managed and shared amongst stakeholders. The information management includes data across biological scales, from individual molecules, molecular patterns, cells and tissue types, organs, clinical phenotypes to individual patient profiles. The information stream includes all stakeholders from translational medicine to decision-making clinicians and treated patients.

The mission of integrative bioinformatics is to provide technological tools enabling collaboration across medical fields, scientific disciplines and geographical barriers in order to fulfil the following tasks:

–Gathering of complex data received from emerging technologies, such as medical imaging, pharmacogenetics, clinical omics, pathology-specific molecular patters, disease modelling and individual patient profiles.

–Undertaking both retrospective and prospective analyses of biodata, information and knowledge related to the (epi-) genome and its links to disease for translational medicine.

–Providing help with information and knowledge to advance health-related sciences and to make health care services more reliable.

–Analysing complex technological inputs for making optimal clinical decisions.

–Learning from what has happened at the individual, process and health system levels to promote the integrative approach by predictive, preventive and personalised medicine (PPPM).

–Creating patient records and securing safety treatment of patient databases.

–Promoting standardisation in health care.

–Presenting integrated data innovatively to enhance comprehension.

### Management of the sensitive ethical aspects linked to the patient records

From what was mentioned previously, it is getting clear that the innovative technologies of multidisciplinary analysis summarised by comprehensive patient records provide the complete information about individual health- and pathology-specific profiles as well as about potential disease predispositions of the individual. Therefore, the concomitant professional activities should be dedicated to the ethical issues, namely

–Secure the rights of the patient.

–Secure the strict control over but also the rights of the specialised PPPM centres.

–Clearly define the authorisation to access individual records in the database.

–Clearly define the authorisation to interpret individual data.

–Secure and guarantee the access to the database for authorised professionals only.

–Regularly analyse potential long-term consequences of the recorded data: health benefits for patients versus potential danger of data misuse and wrong interpretation.

–If necessary, revise the juristic platform to satisfy the needs of patients properly.

A high quality of ethical standards is the prerequisite for the successful implementation of PPPM in health care systems [20,33].

### Advancing ‘participatory medicine’: effective promotion of health knowledge and health language in the general population and collaboration with organised patient groups

‘Nothing about me without me’ is an excellent slogan of the Society of Participatory Medicine [34] which certainly should be broadly accepted by advanced medical services. A number of independent evidence-based studies have demonstrated that the efficacy of treatments strongly depends on the level of harmony in ‘doctor-patient’ collaboration. In order to create the optimal condition for the doctor-patient collaboration, a common language should be used. For that high didactic quality educational measures aiming at significant improvements of health knowledge and health language in the general population are strongly recommended. Unfortunately, information retrieved from the Internet is frequently of poor professional quality providing controversial data that confuse the understanding and slow down the learning process of laymen. People need to be advised of reliable information sources that are well adapted to a corresponding level of understanding (categories of children, youth and adults) and concrete interests of subpopulations (level of education, groups of professionals, patient cohorts). In the field of education, up-to-date information should be accessible to and understandable for the layman on valid predictive and prognostic tests and recommended specialists to be consulted regarding the test interpretation for individual health and disease conditions as well as creation of consequent treatments tailored to the person. These innovations along with the tight collaboration of organised patient groups are one of the strongest instruments of a more effective knowledge promotion [5].

### A new *professional environment* needs a new *culture of communication* of stakeholders for the successful realisation of predictive, preventive and personalised medicine

Taking into account the challenges of current health care systems and paradigm shift from reactive to predictive medicine, a new professional environment should be created to promote research in innovative bio/medical fields and implementation of PPPM in health care. For that, a new professional set-up is needed. However, for their daily communication, different professional groups currently utilise different professional ‘languages’, as a consequence of the ‘classical’ domain-relevant education less understandable for others. This frequently leads to misunderstandings when a great innovation proposed by one professional group is underestimated (or even not valued) by the others, resulting in delays in the implementation across diverse areas in predictive, preventive and personalised medicine. Breaking such communication barriers is a great challenge which can be solved only by international and multidisciplinary networking providing the necessary environment for a creation of multifaceted didactic materials, new programmes specialised in multidisciplinary education and, finally, a new culture of communication of PPPM stakeholders. Herewith, the authors present a consolidated position to the point made as follows: the innovative PPPM-related multidisciplinary educational programmes for professionals should be prioritised in the New European Framework Programme Horizon 2020 as well as by other global and topic-relevant national programmes [4].

In order to promote a new culture of communication of stakeholders in PPPM, worldwide pioneer initiatives have been developed by *The EPMA Journal* focusing on multidisciplinary research in traditional and innovative medical fields. In particular, since 2010 the journal regularly updates both needs and achievements in the field of PPPM applied to common and rare diseases [35]. The following topics are treated by the journal:

–Health care overview in European countries and worldwide.

–New guidelines in health care.

–PPPM applied to chronic non-communicable diseases (diabetes mellitus, cardiovascular disease, cancer and neurodegenerative disorders and others).

–PPPM in rare diseases.

–PPPM in dentistry.

–Predictive medicine in groups at risk.

–Reproductive medicine.

–PPPM in pregnancy, neonatology and paediatrics.

–Body culture, individualised exercise training.

–Nutritional aspects in PPPM.

–Healthy ageing.

–Evidence-based traditional and alternative medicine.

–Integrative bioinformatics, patient records and disease modelling.

–Treatments tailored to the person.

–Innovative drug delivery systems and new therapies.

–Validation of biomarkers.

–Standardisation of innovative biomedical approaches.

–Economic, ethical, legal, social and other relevant aspects of PPPM.

–Collaboration strategies of professional groups with complementary expertise.

## EPMA recommendations

### General report and recommendations in PPPM

**Figure 24:**
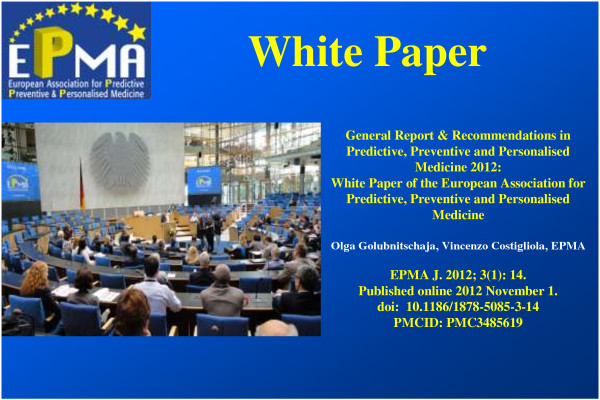
**Recommendations in PPPM.** ‘Recommendations in PPPM’ is the collective product of world-leading experts working in the branches of PPPM under coordination of EPMA. The general report has been prepared as the consortial document proposed at the EPMA World Congress 2011 which took place in Bonn, Germany. This forum has analysed overall deficits and trends relevant for the top-science and daily practice in PPPM focused on the patient.

EPMA has released the package of recommendations for their consideration by research units, educators, health care industry, policymakers, and funding bodies to cover current knowledge deficits in the field and to introduce integrative approaches for advanced diagnostics, targeted prevention, treatments tailored to the person and cost-effective health care. This fundamental document has been published as the EPMA White paper*—*see Figure 24[5].

### Consolidation of professional groups involved in PPPM-relevant research and health care services

#### Status quo

Currently, any multidisciplinary meeting and collaboration of professional groups related to health care usually are results of an enthusiastic initiative of single groups who realise that a multidisciplinary consolidation is the only way on how to advance actual knowledge and services.

#### Recommended approach

Consolidation of professional groups involved in PPPM-related research and health care services is a multifunctional task which should be performed at several levels of activities:

–Mandated optimal set-up of multidisciplinary stakeholders in diagnostics and in the prevention and treatment of individual pathologies.

–Regular meetings of stakeholders with multidisciplinary expertise motivated and regulated by health care-related national and international programmes.

–Multidisciplinary education as an obligation for related professional groups.

–Creation of optimal economic conditions motivating a multidisciplinary expertise to be applied in health care sciences and services.

### Essential measures to implement PPPM concepts

#### A ‘trans-disciplinary’ coordinating and networking action is recommended

Implementation of the innovative PPPM concepts in health care is a 3-dimensional challenge necessitating

1. International coordination, networking and dissemination as a global approach for combating major pathologies and improving economy of health care.

2. Coordination, networking and dissemination for more effective treatment of collateral pathologies (e.g. frequent cardiovascular complications occur in patients with a history of diabetes).

3. Coordination, networking and dissemination among stakeholders (individual professional groups involved in disease-related science and management).

Within the ‘trans-domain’ action, for structuring the work and transparency of the action, individual professional domains may be grouped into two categories of primary expertise (individual domains): (A) ‘General ’ and (B) ‘Technological tools’.

A. General domains represent following professional groups and activities: Development of PPPM concepts in health care, Integrative Bioinformatics, Translational Medicine, Traditional, Complementary and Alternative Medicine, TCAM, Health care economy, Medical Ethics, Evidence-based assessment of health, Food, Well-being, Professional integration in personalised medicine, Integrative Education in PPPM, Promotion of young professionals, Creation of innovative PPPM-relevant programmes for research and implementation, Dissemination.

B. Technological tools are specialised professional activities in Laboratory Medicine, Medical Imaging, Predictive Diagnostics, Biopreservation and Biobanking, Disease Modelling, and Patient Profiling, Targeted Prevention, Gender Medicine, Clustering of Specialised Centres, Validation and Standardisation, Patenting and Industrial Products, Evaluation of Clinical Application.

**Figure 25:**
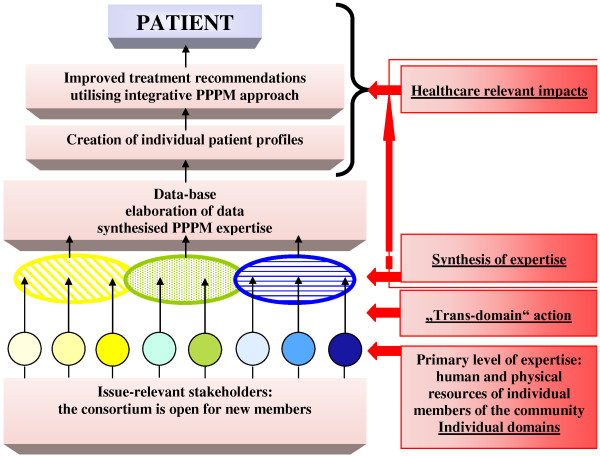
**General scheme recommended for the implementation in the trans-disciplinary-action.** Main deliverable and health care-relevant impacts are summarised utilising the synthesised PPPM expertise of individual domains relevant for predictive, preventive and personalised medicine in health care.

A synthesis of professional domains is highly recommended as the trans-domain action to address current deficits in PPPM-relevant professional knowledge and health care. For that, well-designed coordinating measures, data dissemination and networking among bio/medical scientists, research centres, health care providers, industrial partners, educators, patient organisations, policymakers, regulatory, funding and publishing bodies, and the public domain are essential to be performed according to the general scheme shown in Figure 25.

### Integrative education is highly recommended for PPPM specialists

The coordination in ‘Integrative Education in PPPM’ should foresee following measures:

•A deep analysis of current barriers and actions needed to implement multidisciplinary programmes in PPPM, taking into account the existence of specific directives for medical education and the short list of recognised medical specialties.

•A consequent creation of the PPPM-specific ‘Communication Units’ to analyse the deficits in multidisciplinary communication and to coordinate the measures aimed at addressing these deficits, both in the short-term (bridging the acute interdisciplinary communication) and in the long-term (information output for educational programmes) ones.

•Recruiting PPPM multidisciplinary taxonomy for educational purposes.

•Elaboration of the didactic approach and recommendations for preparing a series of ‘White papers’ and ‘Position papers’ dedicated to the innovative concepts of PPPM and input to standardisation bodies, policymakers, regulators and users.

•Establishing innovative initiatives in the education of professionals to identify main concepts and competence frameworks bridging separate fields of science/disciplines to achieve breakthroughs that require an interdisciplinary approach.

•Describing technological scenarios for learning that might be useful to build spaces where knowledge can be constructed collaboratively.

•Based on the experience from the performance of the above measures, a creation of new well-designed university curricula on PPPM.

### Standardisation of health care services in Europe: how to proceed

This is a strategic issue for policymakers after detailed consultations with the relevant health care professionals. In order to effectively promote advanced medical services, much more harmonisation among stakeholders is needed across the Europe. The process of harmonisation requires clear focuses and should result in reasonable standards introduced for comparable/analogous functional elements and systems. All of the existing systems should be carefully analysed at the European level before corresponding standards may be elaborated and introduced in daily medical practice.

The European standards recommended by experts, in particular, for analytical systems are as follows [20]:

•Consistent standards of laboratory (any other analytical) practice.

•Conformance with all relevant international standard systems.

•Equivalent units of measurement.

•Traceability of the measures.

•Equal quality of measurements.

•Equal reference systems.

•Equal evaluation systems.

The European standards recommended for the European electronic patient record are as follows:

–Mandated multidisciplinary examination approach.

–Unified communication protocols for data exchange between different information systems in health care.

–Consolidated algorithms.

–Standardised and complex interpretation of analytical results.

It is obvious that these listed measures are tasks superior to the national level. This may be effectively achieved, if corresponding European resources will be adequately mobilised, including consolidated professional efforts and dedicated budgets.

## Summary and outlook

The new European programme Horizon 2020 is certainly not a hype but a hope for the optimistic scenarios in health care research and implementation, which Predictive, Preventive and Personalised Medicine creates a robust platform for. Horizon 2020 places to the disposal of professionals the long-lasting instruments for scientific and technological progress in medical services and health care-related programmes. However, the overall success of the programme strongly depends on the effective communication and consolidation of all the stakeholders relevant for PPPM research and implementation as well as the communication quality between health care providers and policymakers followed by the most optimal political decision in favour of an active PPPM realisation in health care sector.

Since 2008, EPMA considers acute problems in medical sciences as well as the quality and management of medical services challenging health care systems in Europe and worldwide. The Association is focused on the creation of the long-term strategies and professional consolidation to promote PPPM-related innovation in research and health care. Using a well-consolidated expertise, EPMA has contributed with the evidence-based scientific PPPM protocols to the contents of Horizon 2020—the facts that EPMA members are much proud about. Consequently, a series of the PPPM-relevant projects of the EPMA experts and our partners in Europe and worldwide are currently under consideration, preparation and submission procedure that follows already the very first calls released by the EU Commission within the Horizon 2020 for the years 2014–2015. The projects are focused on the patients' needs, innovative medical sciences, optimal health and disease management, coordinating and networking measures, expert recommendations for the relevant medical fields and optimal solutions which have a potential to advance health care services if the long-term strategies were to be effectively implemented as elaborated and published in the scientific works and fundamental documents of the EPMA consortia.

Also in the future, EPMA will consequently follow the fundamental principles of the Association such as the full transparency of the global strategies, permanent information stream towards the stakeholders and best possible consolidation of the professionals in PPPM. Over the entire lifetime of the Horizon 2020, EPMA will permanently update the world with the regularly published position paper which will inform about the PPPM-relevant projects submitted and evaluated to the individual topics/call and the European programme as a whole.

## Competing interests

The authors declare that they have no competing interests.

## Authors’ contributions

OG, JK and VC have drafted the manuscript and circulated it with the members of the Association in order to finalise the EPMA position. All authors read and approved the final manuscript.

## Authors’ information

The article is published on behalf of the European Association for Predictive, Preventive and Personalised Medicine and expresses the consolidated position of the EPMA members and representatives.

## References

[B1] Advances in Predictive, Preventive and Personalised Medicine [http://www.springer.com/series/10051]

[B2] Golubnitschaja O Time for new guidelines in advanced diabetes care: paradigm change from delayed interventional approach to predictive, preventive & personalized medicine EPMA J 2010 1 3 12 10.1007/s13167-010-0014-5 23199036 PMC3405298

[B3] Norstedt I Horizon 2020: European perspectives in healthcare sciences and implementation EPMA J 2014 5 A1 10.1186/1878-5085-5-S1-A1

[B4] Golubnitschaja O Swanton C Danesi R Costigliola V Promoting predictive, preventive and personalised medicine: European event of global importance EPMA J 2011 2 131 136 23199141 10.1007/s13167-011-0088-8 PMC3405384

[B5] Golubnitschaja O Costigliola V EPMA General Report & Recommendations in Predictive, Preventive and Personalised Medicine 2012: White Paper of the European Association for Predictive, Preventive and Personalised Medicine EPMA J 2012 1 1 14 23116135 10.1186/1878-5085-3-14 PMC3485619

[B6] EPMA Summit and World Congress 2013 in Brussels: European Association for Predictive, Preventive and Personalised Medicine develops a new culture in communication amongst stakeholders [http://www.epmanet.eu/images/stories/pdfs/EPMA%20Summit%20and%20World%20Congress%202013_press%20release_Oct%202013.pdf]

[B7] The EU Framework Programme for Research and Innovation - health, demographic change and wellbeing [http://ec.europa.eu/programmes/horizon2020/en/h2020-section/health-demographic-change-and-wellbeing]

[B8] The EU Framework Programme for Research and Innovation - What is Horizon 2010? [http://ec.europa.eu/programmes/horizon2020/en/what-horizon-2020]

[B9] Innovation Unit - a Europe 2020 Initiative [http://ec.europa.eu/research/innovation-union/index_en.cfm]

[B10] Golubnitschaja O Yeghiazaryan K Opinion controversy to chromium picolinate therapy's safety and efficacy: ignoring “anecdotes” of case reports or recognising individual risks and new guidelines urgency to introduce innovation by predictive diagnostics? EPMA J 2012 3 11 10.1186/1878-5085-3-11 23039227 PMC3515400

[B11] Golubnitschaja O Mozaffari MS Three levels of prediction, prevention & individualised treatment algorithms to advance diabetes care: integrative approach New Strategies to Advance Pre/Diabetes Care: Integrative Approach by PPPM 2013 Dordrecht, Heidelberg, New York, London: Springer 15 28 ISBN 978-94-007-5970-1

[B12] Golubnitschaja O Golubnitschaja O Diabetes mellitus Predictive Diagnostics and Personalized Treatment: Dream or Reality? 2009 New York: Nova Science 147–150, ISBN 978-1-60692-737-3

[B13] Golubnitschaja O Yeghiazaryan K Costigliola V Trog D Braun M Debald M Kuhn W Schild HH Risk assessment, disease prevention and personalised treatments in breast cancer: is clinically qualified integrative approach in the horizon? EPMA J 2013 4 6 10.1186/1878-5085-4-6 23418957 PMC3615949

[B14] Golubnitschaja O Yeghiazaryan K Cebioglu M Morelli M Herrera-Marschitz M Birth asphyxia as the major complication in newborns: moving towards improved individual outcomes by prediction, targeted prevention and tailored medical care EPMA J 2011 2 197 210 10.1007/s13167-011-0087-9 23199149 PMC3405378

[B15] Yeghiazaryan K Flammer J Golubnitschaja O Predictive molecular profiling in blood of healthy vasospastic individuals: clue to targeted prevention as personalised medicine to effective costs EPMA J 2010 1 263 272 10.1007/s13167-010-0032-3 23199064 PMC3405317

[B16] Golubnitschaja O Yeghiazaryan K Flammer J Key molecular pathways affected by glaucoma pathology: is predictive diagnosis possible? EPMA J 2010 1 237 244 10.1007/s13167-010-0031-4 23199062 PMC3405318

[B17] Yeghiazaryan K Flammer J Golubnitschaja O Mandel S Individual predispositions in healthy vasospastic individuals: patient profiling for targeted prevention of “down-stream” pathologies as cost-effective personalised medicine Neurodegenerative Diseases: Integrative PPPM Approach as the Medicine of the Future 2013 Dordrecht, Heidelberg, New York, London: Springer 13 29 ISBN 978-94-007-5865-0

[B18] Golubnitschaja O Yeghiazaryan K Wunderlich K Schild HH Flammer J Disease proteomics reveals altered basic gene expression regulation in leukocytes of normal-tension and primary open-angle glaucoma patients Proteomics Clin Appl 2007 1 1316 1323 10.1002/prca.200700150 21136628

[B19] Golubnitschaja O Neurodegeneration: accelerated ageing or inadequate healthcare? EPMA J 2010 1 211 215 10.1007/s13167-010-0030-5 23199059 PMC3405328

[B20] Golubnitschaja O Watson ID Topic E Sandberg S Ferrari M Costigliola V Position paper of the EPMA and EFLM: a global vision of the consolidated promotion of an integrative medical approach to advance health care EPMA J 2013 4 12 10.1186/1878-5085-4-12 23663422 PMC3700840

[B21] HORIZON 2020 - WORK PROGRAMME 2014 – 2015 [http://ec.europa.eu/research/participants/data/ref/h2020/wp/2014_2015/main/h2020-wp1415-health_en.pdf]

[B22] Research & Innovation - Horizon 2020 Funding [https://ec.europa.eu/research/participants/portal/desktop/en/home.html]

[B23] Costigliola V Gahan P Golubnitschaja O Golubnitschaja O Predictive medicine as the new philosophy in health care Predictive Diagnostics and Personalized Treatment: Dream or Reality 2009 New York: Nova Science 1 3 ISBN 978-1-60692-737-3

[B24] Golubnitschaja O Costigliola V Changing long-held beliefs is never easy: a proposal for multimodal approaches in female healthcare - an integrative view Healthcare Overview: New Perspectives 2012 Dordrecht, Heidelberg, New York, London: Springer 251 268 ISBN 978-94-007-4602-2

[B25] Costigliola V Costigliola V Mobility of medical doctors as an attribute of the cross-border healthcare: challenges, opportunities and perspectives Healthcare Overview: New Perspectives 2012 Dordrecht, Heidelberg, New York, London: Springer 233 244 ISBN 978-94-007-4602-2

[B26] Carlsen S Petersen PH Skeie S Skadberg Ø Sandberg S Within-subject biological variation of glucose and HbA(1c) in healthy persons and in type 1 diabetes patients Clin Chem Lab Med 2011 49 1501 1507 21631391 10.1515/CCLM.2011.233

[B27] Kapalla M Matuskova D Golubnitschaja O Information systems as an essential component of prediction in the laboratory diagnostics Predictive Diagnostics and Personalized Treatment: Dream or Reality 2009 New York: Nova Science 529 548

[B28] Mandel S Golubnitschaja O Mandel S Time for new guidelines in handling of neurodegenerative disorders: optimistic versus pessimistic prognosis by application of PPPM Neurodegenerative Diseases: Integrative PPPM Approach as the Medicine of the Future 2013 Dordrecht, Heidelberg, New York, London: Springer 3 9

[B29] Shapira N Costigliola V A gender-specific nutritional approach to women's healthcare Healthcare Overview: New Perspectives 2012 Dordrecht, Heidelberg, New York, London: Springer 269 305

[B30] Marcus-Kalish M Costigliola V Simultaneous systematic approach to enable predictive, preventive and personalised medicine - women healthcare as a case study Healthcare Overview: New Perspectives 2012 Dordrecht, Heidelberg, New York, London: Springer 313 331

[B31] Drucker E Krapfenbauer K Pitfalls and limitations in translation from biomarker discovery to clinical utility in predictive and personalised medicine EPMA J 2013 4 7 10.1186/1878-5085-4-7 23442211 PMC3599714

[B32] Kapalla M Niederlag W, Lemke HU, Golubnitschaja O, Rienhoff O Healthcare information complexity and the role of informatics in predictive, preventive and personalized medicine Personalisierte Medizin 2010 Dresden: Health Academy 83 108

[B33] Gefenas E Cekanauskaite A Tuzaite E Dranseika V Characiejus D Costigliola V New Ethical Paradigm in Preventive, Predictive and Personalised Medicine Healthcare Overview: New Perspectives 2012 Dordrecht, Heidelberg, New York, London: Springer 471 484 ISBN 978-94-007-4602-2

[B34] Society for Participatory Medicine [http://participatorymedicine.org/]

[B35] The EPMA Journal [http://www.epmajournal.com/]

